# Altered H3K4me3 profile at the *TFAM* promoter causes mitochondrial alterations in preadipocytes from first-degree relatives of type 2 diabetics

**DOI:** 10.1186/s13148-023-01556-z

**Published:** 2023-09-07

**Authors:** Michele Longo, Federica Zatterale, Rosa Spinelli, Jamal Naderi, Luca Parrillo, Pasqualina Florese, Cecilia Nigro, Alessia Leone, Augusta Moccia, Antonella Desiderio, Gregory A. Raciti, Claudia Miele, Ulf Smith, Francesco Beguinot

**Affiliations:** 1 Department of Translational Medicine, Federico II University of Naples, Naples, Italy; 2 URT Genomics of Diabetes, Institute of Experimental Endocrinology and Oncology, National Research Council, Naples, Italy; 3 Lundberg Laboratory for Diabetes Research, Department of Molecular and Clinical Medicine, Sahlgrenska Academy, University of Gothenburg, Göteborg, Sweden

**Keywords:** Type 2 diabetes, Adipose tissue dysfunction, Epigenetic mechanisms, Mitochondrial alterations, Adipogenesis, Premature senescence

## Abstract

**Background:**

First-degree relatives of type 2 diabetics (FDR) exhibit a high risk of developing type 2 diabetes (T2D) and feature subcutaneous adipocyte hypertrophy, independent of obesity. In FDR, adipose cell abnormalities contribute to early insulin-resistance and are determined by adipocyte precursor cells (APCs) early senescence and impaired recruitment into the adipogenic pathway. Epigenetic mechanisms signal adipocyte differentiation, leading us to hypothesize that abnormal epigenetic modifications cause adipocyte dysfunction and enhance T2D risk. To test this hypothesis, we examined the genome-wide histone profile in APCs from the subcutaneous adipose tissue of healthy FDR.

**Results:**

Sequencing-data analysis revealed 2644 regions differentially enriched in lysine 4 tri-methylated H3-histone (H3K4me3) in FDR compared to controls (CTRL) with significant enrichment in mitochondrial-related genes. These included *TFAM,* which regulates mitochondrial DNA (mtDNA) content and stability. In FDR APCs, a significant reduction in H3K4me3 abundance at the *TFAM* promoter was accompanied by a reduction in *TFAM* mRNA and protein levels. FDR APCs also exhibited reduced mtDNA content and mitochondrial-genome transcription. In parallel, FDR APCs exhibited impaired differentiation and *TFAM* induction during adipogenesis. In CTRL APCs, *TFAM*-siRNA reduced mtDNA content, mitochondrial transcription and adipocyte differentiation in parallel with upregulation of the *CDKN1A* and *ZMAT3* senescence genes. Furthermore, *TFAM*-siRNA significantly expanded hydrogen peroxide (H_2_O_2_)-induced senescence, while H_2_O_2_ did not affect *TFAM* expression.

**Conclusions:**

Histone modifications regulate APCs ability to differentiate in mature cells, at least in part by modulating *TFAM* expression and affecting mitochondrial function. Reduced H3K4me3 enrichment at the *TFAM* promoter renders human APCs senescent and dysfunctional, increasing T2D risk.

**Graphical abstract:**

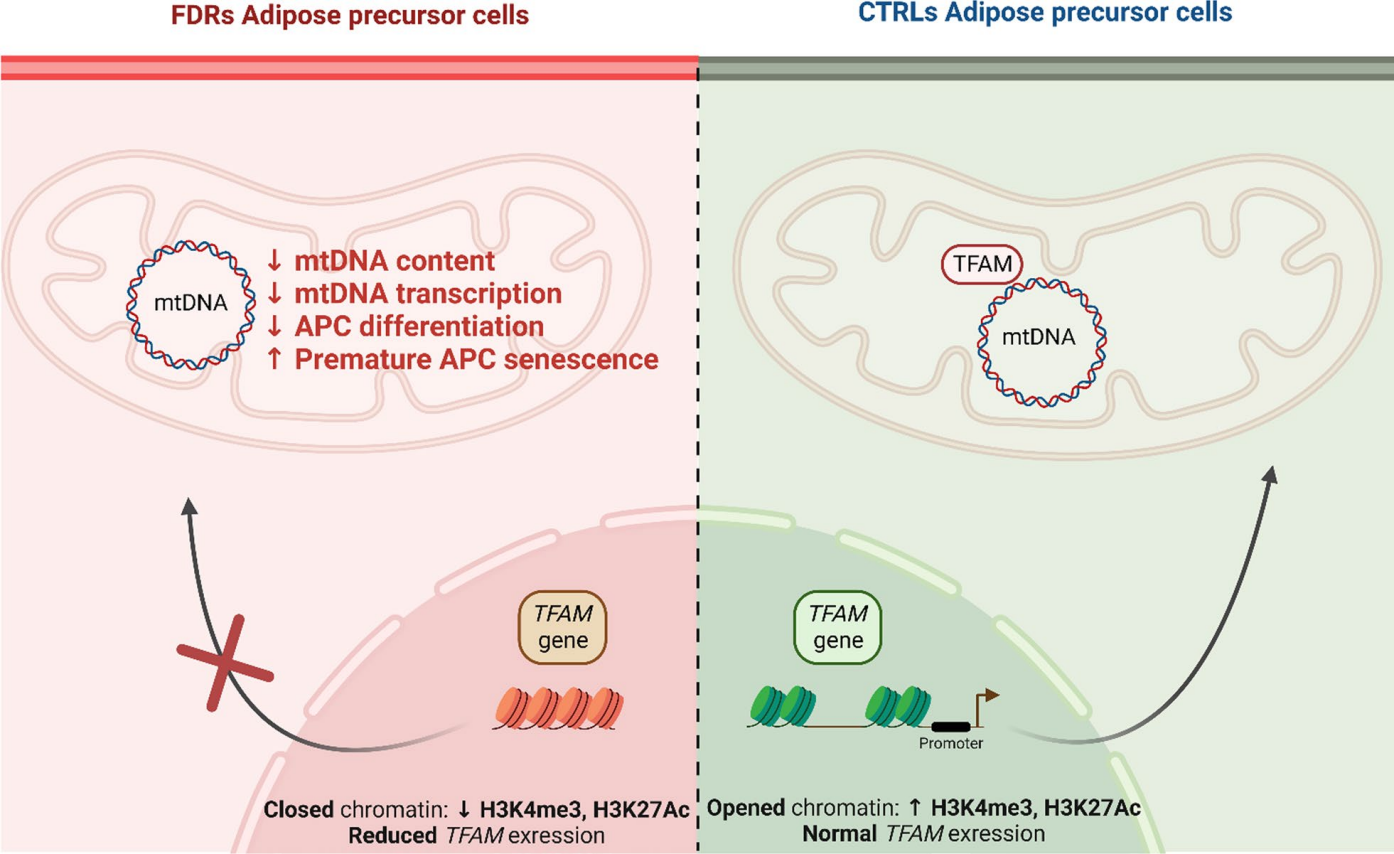

**Supplementary Information:**

The online version contains supplementary material available at 10.1186/s13148-023-01556-z.

## Introduction

Mitochondrial abnormalities and dysfunctions in metabolically relevant tissues, such as adipose tissue, have long been recognized in obesity and type 2 diabetes [1, 2; T2D]. In obesity, increased plasma triacylglycerol levels and peripheral tissue accumulation lead to lipotoxicity, oxidative stress, impaired oxidative phosphorylation, and substrate metabolism [3, 4]. In T2D, mitochondrial dysfunction accompanies insulin resistance, marking progression toward diabetes [1, 2]. Whether mitochondrial dysfunction primarily develops in obesity, which then contributes to the evolution toward T2D, or whether it precedes diabetes debut independent of obesity remains unclear though.

Dysfunctional adipose tissue is critical to developing chronic low-grade inflammation, insulin resistance and progression toward T2D, as preservation of adipocyte plasticity mitigates the effects of the caloric overflow, preserving peripheral insulin sensitivity and metabolic health [4, 5]. Indeed, metabolically obese but normal-weight subjects commonly feature a high risk of T2D development, regardless of their body mass index (BMI), as originally recognized by Ruderman et al. [6–8].

Healthy first-degree relatives of type 2 diabetics (FDR) exhibit subcutaneous adipose tissue (SAT) early senescence, inflammation and hypertrophy, independent of obesity and body fat distribution [9–12]. These alterations occurred long before the onset of T2D and were shown to be central in the progression toward T2D [11–14]. FDR individuals also show a risk of developing T2D up to tenfold higher compared to age-matched subjects without a T2D family history, depending on the number of affected relatives [15–17]. In FDR individuals, early senescence, insulin resistance, impaired adipogenesis, dysfunctional adipose tissue, and the risk of T2D are closely associated with hypertrophic SAT and are detectable even when glucose tolerance is preserved [9–11, 13, 18–20]. SAT hypertrophy in these subjects is caused by impaired adipocyte precursor cells (APCs) recruitment into the adipogenic program rather than by a deficiency of APCs [21–23]. In SAT hypertrophy of FDR, the white adipose tissue (WAT) becomes critically dysfunctional and does not expand appropriately in response to excess caloric overload. Limited storage capacity of the SAT causes ectopic fat deposition in different major organs contributing to glucose homeostasis leading to lipotoxicity and worsening systemic insulin-resistance and an excess risk of T2D [4, 24, 25]. Despite intensive investigation, the detailed molecular mechanisms responsible for the impaired SAT-APCs adipogenesis in FDR individuals have been only partially elucidated [12, 21, 26–28].

Epigenetic modifications mediate, to a large extent, the environmentally induced excess risk of T2D [29, 30]. Epigenetics play a direct role in multiple crucial stages of adipogenesis, from the commitment of multipotent precursor cells to terminal adipocyte differentiation [31, 32]. It has further been shown that SAT hypertrophy in FDR subjects is, at least in part, caused by changes in the methylation status at several DNA loci and by altered expression of specific microRNAs in APCs isolated from SAT [27, 28]. Different from DNA methylation and microRNAs expression, the involvement of histone modifications in SAT hypertrophy in FDR individuals has never been investigated, while their contribution to the molecular mechanisms, which regulate APCs commitment and differentiation, has long been demonstrated [32–34].

The present study particularly aimed at identifying changes in the profile of genome-wide trimethylation at lysine 4 on histone H3 (H3K4me3) in adipose progenitor cells isolated from SAT of healthy FDR and control matched subjects with no T2D familiarity. H3K4me3 is an important signal determining cell identity and significantly contributes to stem cell biology [35]. Therefore, we have explored its significance to restricted adipogenesis and T2D risk in subjects who are FDR of type 2 diabetics. Our work reveals that these subjects feature characteristic changes in the H3K4me3 profile at the promoter of the *mitochondrial transcription factor A* (*TFAM*) gene, crucial for mitochondrial DNA (mtDNA) replication and mitochondrial-encoded gene transcription [36]*.* Its reduced mRNA expression decreases mtDNA content and mitochondrial transcription and impairs APCs differentiation in FDR individuals. In addition, we further implicate mitochondrial dysfunction in the development of premature senescence in APCs from FDR individuals.

## Materials and methods

### Study participants

Twenty subjects were chosen from the European Network on Functional Genomics of Type 2 Diabetes (EUGENE2) study population [37]. These individuals were healthy, non-obese, and either had one first-degree relative with T2D (FDR; *n* = 9) or no diabetes familiarity (CTRL; *n* = 11). The study received ethical approval (approval numbers: S655-03 and T492-17) from the Ethical Committee of the Sahlgrenska Academy, University of Gothenburg, in line with the principles of the Helsinki Declaration. All participants provided informed consent prior to the enrollment in the study.

### APCs isolation, culture, differentiation, and treatment

Abdominal SAT biopsies were collected via needle aspiration from the periumbilical area. The biopsies were subjected to collagenase digestion (concentration of 0.9 mg/mL) for 45 min at 37 °C, followed by filtration through a 250-μm nylon mesh. The resulting cell pellet, consisted of cells from the stromal vascular fraction including APCs, was collected. To remove erythrocytes, the cell pellet was washed twice with a solution of 155 mmol/L NH4Cl for 5 min. Following a 3-day incubation period, the remaining CD14 + and CD45 + inflammatory cells and CD31 + endothelial cells were eliminated by magnetic immune separation. APCs were then maintained in a medium composed of a mix of Dulbecco's modified Eagle medium (DMEM) and Ham's F12 (F12) (Lonza, Basel, Switzerland; 31330-038) supplemented with 10% fetal bovine serum (FBS) (ThermoFisher Scientific, Waltham, MA, USA; 10270–106), 2 mmol/L glutamine (Euroclone, Milan, Italy; ECB3000D), 100 units/mL penicillin and 100 μg/mL streptavidin (Lonza, Basel, Switzerland; 10140-122). Following an additional 3-day incubation period, the remaining inflammatory and endothelial cells were eliminated by immune magnetic separation, as previously described [9, 22].

To induce adipocyte differentiation, the cells were cultured until reaching confluence in a medium supplemented with 10% FBS. After 3 days of confluence, the cells were grown in DMEM/F12 medium supplemented with a differentiation mix consisting of 850 nmol/L insulin (Humulin, Eli Lilly, HI0210), 10 μmol/L dexamethasone (Sigma-Aldrich, St Luis, MO, USA; 04902), 0.5 mmol/L isobutylmethylxanthine (MP Biomedicals, Irvine, CA, USA;195262), 10 μmol/L rosiglitazone (Sigma-Aldrich, St Luis, MO, USA; R2408), 2 mmol/L glutamine, 3% FBS and antibiotics. Following 3 days, the medium was exchanged with a fresh medium containing 850 nmol/L insulin, 1 μmol/L dexamethasone, 1 μmol/L rosiglitazone, 2 mmol/L glutamine and 10% FBS. Then, the cells were enabled to differentiate with the culture medium changed every 3 days [22].

To assess lipid accumulation, the differentiated APCs were stained with Oil Red O. Mature adipocytes were fixed in 4% formaldehyde and incubated in the Oil Red O staining solution for 60 min (Sigma-Aldrich, St. Louis, MO, USA; O0625). The Olympus microscope was utilized for images acquisition (Olympus, Center Valley, PA, USA). To quantify lipid accumulation, the Oil Red O dye was extracted in isopropyl alcohol, and the absorbance at 490 nm was assessed by a spectrophotometer (Beckman, Los Angeles, CA, USA).

To induce senescence, CTRL APCs were exposed for 1 h to 200 µmol/L hydrogen peroxide (H_2_O_2_; Sigma-Aldrich, St Luis, MO, USA; H1009). FDR APCs were treated with a combination of the senolytic drugs Dasatinib (Sigma-Aldrich, CDS023389) and Quercetin (Sigma-Aldrich, 04951). For 3 days, the cells were exposed to 0.5 μmol/L Dasatinib plus 20 μmol/L Quercetin or vehicle only (DMSO).

Cellular ATP content was measured using the ATP luminescent assay kit (Cayman, Ann Arbor, MI, USA; 700410) in accordance with the manufacturer's instructions.

### ChIP-qPCR and ChIP-Seq assay

1 × 10^5^ cells were fixed for 8 min with 1% formaldehyde, followed by quenching the reaction with 125 mmol/L glycine for 5 min. Lysis buffer (SDS 1%, 10 mM/L EDTA, 50 mM/L Tris–HCL, pH 8.1) was added to the pellet and incubated for 5 min on ice. The cross-linked chromatin was sonicated using the Bioruptor® sonicator (Diagenode, Seraing, Belgium). Cellular debris was eliminated through centrifugation. Chromatin was immunoprecipitated using anti-H3K4me3 (Diagenode, C15200152), H3K27ac (Histone H3 lysine 27 acetylation) (Diagenode, C15410196), and H3K27me3 (Histone H3 lysine 27 trimethylation) (Diagenode, C15410195) antibodies and with anti-mouse IgG beads as negative control (Thermo Fisher Scientific, Waltham, MA, USA; sc 2025). The 10% of chromatin was recovered and used as INPUT sample. The immunoprecipitated fragments were then washed and eluted at 37 °C for 1 h with the elution buffer (SDS 1% e NaHCO_3_ 0,1 M) before being de-cross-linked overnight with 5 mol/L NaCl at 65 °C. Proteins were eliminated using 1 mol/L proteinase K, 0.08 mol/L Tris–HCl, and 0.01 mol/L EDTA for 1 h at 45°. The immunoprecipitated DNA was purified using QIAquick PCR Purification Kit (Qiagen, Hilden, Germany; 28106) following the instructions provided by the manufacturer. Chromatin immunoprecipitation (ChIP) enrichment was quantified by quantitative real-time PCR (qPCR) using specific primers (for primer sequences, see Additional file 1: Table S1). ChIP enrichments were calculated as a percentage relative to the input DNA (10%) using the following equation: 2^[(Ct INPUT − log (*X*%)/log2) − *Ct* sample] × 100%, where [log (*X*%)/log2)] accounts for the INPUT dilution 1/X.

Four study participant pools for the Chromatin Immunoprecipitation Sequencing (ChIP-Seq) assay were prepared accordingly to the aforementioned protocol. Both immunoprecipitated and INPUT samples were sent to IGA Technology Services, and ‘Ovation Ultralow Library System v2’ kit (NuGEN, San Carlos, CA, USA) has been adopted for library preparation following the manufacturer’s instructions. Quantification of both INPUT and immunoprecipitated samples, as well as the final libraries, was performed using the Qubit 2.0 Fluorometer from Invitrogen (Carlsbad, CA, USA) and quality-tested by Agilent Bioanalyzer High Sensitivity DNA assay (Agilent Technologies, Santa Clara, CA, USA) and Real Time Stratagen Mx3000P (Agilent Technologies, Santa Clara, CA, USA). Libraries were then prepared using Illumina cBot for cluster generation on the flowcell, following the guidelines provided by the manufacturer and sequenced on paired-end 125-bp mode on HiSeq2500 (Illumina, San Diego, CA, USA). The CASAVA 1.8.2 version of the Illumina pipeline was used to process raw data for both format conversion and de-multiplexing. The ERNE and Cutadapt tools were used to remove low-quality bases and adapters. Trimmed reads were aligned to the human reference genome (human hg19) with Bowtie2 (with default parameters), and uniquely mapping read selection was performed on alignments. Contaminants assessment and examination on how well the signal in the ChIP-Seq sample can be differentiated from the background distribution of reads in the control sample have been done through bamFingerprinting. Peaks were detected with MACS2 (with default parameters), capturing the influence of genome complexity to evaluate the significance of enriched ChIP regions. The qualitative comparison of BED Peak files has been carried out through the BEDTools. The peaks annotation and the identification of the potential association between ChIP regions and functionally important genomic regions were assessed using the online tool PAVIS (https://manticore.niehs.nih.gov/pavis2/). The analysis provides statistics on ChIP enrichment at important genome features. The functional annotation tool Enrichr (https://maayanlab.cloud/Enrichr/#) was adopted to categorize genes exhibiting histone peaks based on cellular component localization.

### *TFAM*-siRNA transfection

APCs were seeded in 6-well plates, and the medium without antibiotics was added, enabling cells to reach 60–80% of confluence. The cells were transfected with 30 pmol/L *TFAM*-siRNA (Ambion™ Silencer™ Select Pre-Designed siRNA; 4392420 s14002) or negative control (scramble) using lipofectamine RNAimax reagent (Invitrogen, Carlsbad, CA, USA; 13778-030). The preparation of the siRNA-lipofectamine mix was carried out in accordance with the guidelines provided by the manufacturer. The cells were then incubated in a humidified incubator at 37 °C and 5% CO_2_ and harvested after 48 h of culture. CTRL APCs were also differentiated and silenced throughout the entire differentiation process, starting with the confluence stage on day 0 and ending with mature adipocytes on day 15. The siRNA-lipofectamine mixture was replaced together with the differentiation mix every 3 days.

### RNA isolation and qPCR

Total RNA was isolated from APCs of CTRL and FDR subjects using EuroGold Trifast reagent in accordance with the instructions provided by the manufacturer (Euroclone, Pero, MI, Italy; EMR517100). RNA samples were reverse-transcribed into complementary DNA (cDNA) by SuperScript III Reverse Transcriptase from Life Technologies (San Diego, CA, USA; 18080-044). The resulting cDNA served as the template for the subsequent qPCR analysis, conducted using the iTAQ Universal SYBR Green Supermix (Bio-Rad, Hercules, CA, USA; 1725124) on QuantStudio 7 Flex system (Applied Biosystems, Foster City, CA, USA).

Data normalization was achieved using the housekeeping gene *Ribosomal protein L13a* (*RPL13A*). Relative quantification of gene expression was determined using the comparative 2 − ∆∆*Ct* method, based on the cycle threshold (*Ct*) values of the target and housekeeping genes. Absolute quantification (Absolute Units, AU) of gene expression was calculated using the comparative 2 − ∆*Ct* method and shown as individual data points. The primer sequences utilized for the analysis, indicated in Additional file 1: Table S1, were designed using the Primer-Blast online tool (http://www.ncbi.nlm.nih.gov/tools/primer-blast/). The primer sequences were obtained from Sigma-Aldrich (St Louis, MO, USA).

### Genomic DNA extraction, DNA methylation assessment, and mtDNA copy number determination

Genomic DNA was extracted from APCs of FDR and CTRL using a DNA Purification Kit (Promega, Madison, WI, USA; A1120). The resulting DNA underwent bisulphite conversion utilizing the EZ DNA Methylation Kit from Zymo Research (Orange, CA, USA; D5002). The bisulphite-converted DNA was then amplified via PCR using bisulphite-specific primers for *TFAM* gene. The bisulphite genomic sequencing was executed according to the protocol described in our previous publication [38]. Sequencing was carried out on an ABI 3500 Automatic Sequencer utilizing Big Dye Terminator v3.1 (Applied Biosystems, Foster City, CA, USA; 4336917). All procedures were conducted in accordance with the instructions provided by the manufacturer.

The mtDNA content was determined by qPCR, using the mitochondrial *tRNA* (*Leu*) gene normalized to the nuclear *Hemoglobin subunit beta* (*HBB*), as previously described [39]. The mtDNA copy number, relative to nuclear DNA, was determined utilizing the following formulas: Δ*CT* = (nuclearDNA CT—mtDNA CT); Relative mitochondrial DNA content = 2 × 2Δ*CT*.

### Western blot analysis

APCs from FDR and CTRL individuals were dissolved in lysis buffer (20 mmol/L Tris–HCL, pH 7.5, 150 mmol/L NaCl, 10 mmol/L EDTA, 10 mmol/L Na_2_P_2_O_7_, 2 mmol/L Na_3_VO_4_, 100 mmol/l NaF, 1 mmol/L phenylmethylsulfonylfluoride and 10 μg/ml aprotinin). Samples were incubated on ice for 1 h after adding lysis buffer. The Bradford assay was used to assess the protein concentration (Bio-Rad Laboratories, Hercules, CA, USA; 5000001). Protein lysates (50 μg) were subjected to SDS-PAGE and transferred onto a PVDF membrane. Membranes were initially incubated with antibodies targeting TFAM (1:100) (Abcam, Cambridge, UK; Ab170304,) and Vinculin (1:10,000) (Santa Cruz Biotechnology, Dallas, TX, USA; sc-73614) and subsequently with secondary rabbit (1:1000) (Bio-Rad, Hercules, CA, USA; 170-6515) or mouse antibodies (1:10,000) (Bio-Rad, Hercules, CA, USA; 170-6516). Immunoreactive bands were detected by chemiluminescence after incubation with antibodies, and densitometric analysis was performed using ImageJ software (version 1.47t).

### Statistical analysis

The Shapiro–Wilk test was adopted to examine the normal distribution of data. For normally distributed variables, the statistical difference between the means of the experimental conditions was assessed using the two-tailed Student's *t*-test. Not normally distributed data were compared between conditions using the two-tailed Mann–Whitney test. Pearson's and Spearman's rank correlation tests were adopted in correlation analysis to determine statistical significance for normally and non-normally distributed variables, respectively. Significant *p *value is indicated as ***p < 0.001, **p < 0.01 and *p < 0.05. Statistical analyses were conducted by GraphPad Software (v8 for Windows, La Jolla, CA, USA).

## Results

### Differential H3K4me3 enrichment in subcutaneous APCs in FDR and CTRL individuals

To explore whether epigenetic features contribute to the adipocyte abnormalities that increase diabetes risk in FDR of T2D individuals, we have investigated a subgroup of participants to the EUGENE study (Table 1) [37]. Non-obese FDR (*n* = 9) and control subjects (CTRL; *n* = 11) were matched for age, BMI, and body fat percentage. As anticipated based on our previous work [9], these FDR were healthy subject. However, they exhibited larger SAT adipocytes (*p *value < 0.001) and also showed reduced insulin sensitivity (*p *value < 0.01), higher fasting plasma insulin (*p *value < 0.01), increased 2-h plasma glucose after the 75 g oral glucose load (*p *value < 0.05) and glucose levels (*p *value < 0.01).

**Table 1:** Clinical features of FDR and CTRL individuals

Phenotypes	FDR subjects	CTRL subjects	*p *value
*N* (female/male)	9 (5/4)	11 (6/5)	> 0.9999
Age, years	42.33 ± 8.72	38.73 ± 7.76	0.3398
BMI, Kg/m^2^	25.36 ± 1.53	24.46 ± 2.30	0.2221
Fat percent, %	26.89 ± 7.28	23.96 ± 6.42	0.5516
Waist-to-hip ratio (WHR)	0.90 ± 0.05	0.81 ± 0.07	0.0021
FFM, kg	57.13 ± 11.74	57.08 ± 10.43	0.9262
Subcutaneous adipocyte size, μm	100.24 ± 5.08	89.42 ± 6.26	0.0005
f-insulin, pmol/L	60.10 ± 22.71	35.36 ± 12.98	0.0091
fb-glucose, mmol/L	4.82 ± 0.42	4.36 ± 0.40	0.007
OGTT p-glucose 2 h, mmol/L	6.56 ± 1.71	4.84 ± 1.24	0.0346
GIR/bw, mg/kg/min	7.93 ± 1.70	11.50 ± 2.51	0.0023

All data are expressed as mean ± SD, and statistical significance between the two groups was determined by Mann–Whitney test for continuous variables and Fisher's exact test for categorical variable

*BMI* body mass index, *CTRL* subjects with no diabetes familiarity (controls), *f-insulin* fasting insulin, *fb-glucose* fasting blood-glucose, *FDR* first‐degree relatives of T2D subjects, *FFM* fat free mass, *GIR/bw glucose* infusion rate/body weight, *OGTT* p-glucose plasma glucose levels during oral glucose tolerance test

**Fig. 1:**
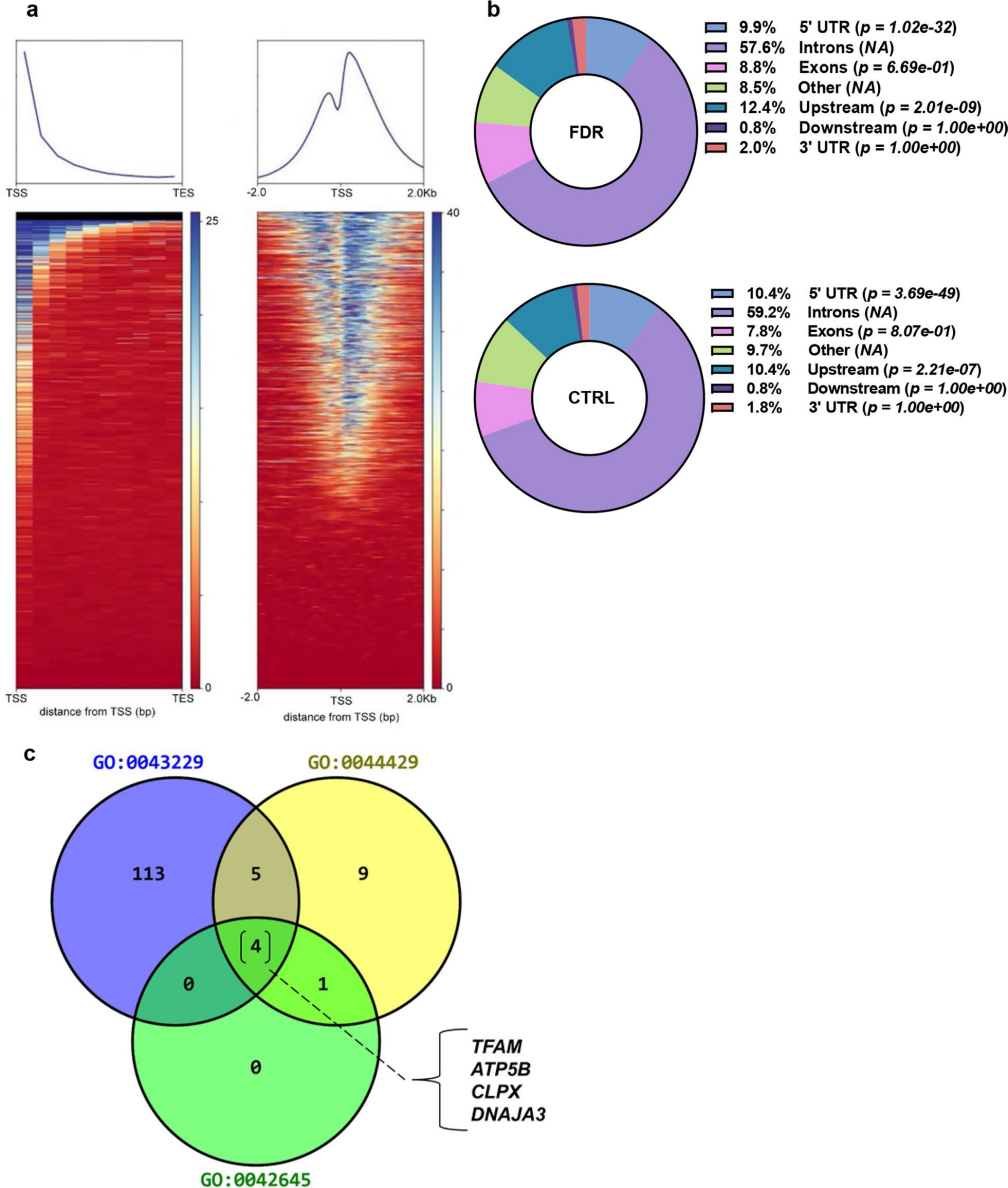
Differential H3K4me3 enrichment in FDR and CTRL subcutaneous preadipocytes. **a** The average genome-wide histone enrichment, calculated by MACS near TSS (± 1000 bp), was measured for all known genes for each individual H3K4me3 and illustrated for individual samples [SVF (blue)]. The relative-fold enrichment was calculated by the MACS algorithm, which was considered for the background signal by comparing the ChIP peaks within an individual study sample to its own 10% INPUT DNA control by looking for reading orientation and mapping density that represent histone (H3K4me3) binding. **b** The pie charts generated from the ChIp-Seq peaks annotation show the distribution of the peaks among the genomic features in CTRL and FDR groups. Peak enrichment test using a binomial *p value* to compare relative peak enrichment across genomic regions of different categories (e.g., upstream promoter region, 5′-UTR, 3′-UTR) is shown in the figure. **c** The figure shows a Venn diagram of the overlapping genes among the three most enriched GO terms

ChIP-Seq profiling of the H3K4me3 mark was then performed in APCs isolated from abdominal SAT biopsies in the FDR and CTRL subjects. The H3K4me3 ab-precipitated chromatin isolated from these individuals was analyzed by deep sequencing, and ChIP-Seq bioinformatic analysis provided the entire H3K4me3 map covering the entire genome. The sequencing-reads were regularly distributed across the human genome and detected in all chromosomes (data not shown). As previously published [40, 41], H3K4me3 enrichment immediately flanks the gene transcription start sites (TSS); the heatmap is shown in Fig. 1a; additional information regarding reads distribution is presented in Additional file 1: Fig. S1a. 2644 H3K4me3 differentially enriched regions (DER) were identified in the CTRL and FDR groups. These regions were similarly distributed in the two groups (1477 CTRL *vs* 1167 FDR; Additional file 1: Fig. S1b). DER were then annotated. Of the 1477 and 1167 DER identified, respectively, in the CTRL and FDR subjects, 1051 and 797 genes were annotated, respectively, in each of the two groups (Additional file 1: Fig. S1c) near the gene TSS (± 1000 bps) and are listed in Additional files 2 and 3, confirming that the H3K4me3 is significantly enriched at the transcription start site regions. The relative enrichment levels at loci in different genomic regions are presented in Fig. 1b. In addition, enrichment analysis on H3K4me3 differentially enriched genes was performed in FDR and CTRL subjects to identify significant enrichment of these genes with respect to specific cellular components (Table 2, Additional file 1: Table S2). Intriguingly, the analysis of cellular components for annotated genes showed a significant enrichment in genes involved in the intracellular organelle (GO:0043229) (*p* value = 0.001700; Fisher’ exact test), mitochondrial part (GO:0044429) (*p* value = 0.002309; Fisher’ exact test), and mitochondrial nucleoid (GO:0042645) (*p *value = 0.002648; Fisher’ exact test), suggesting an involvement of this cellular component in the preadipocytes abnormalities observed in those individuals who are FDR. As shown in Fig. 1c, merging analysis of the overlapping genes belonging to the three most enriched GO terms returns a shortlist of four shared genes.

**Table 2:** GO cellular component analysis

	GO term	*p *value	Odds ratio	Combined score
1	Intracellular organelle (GO:0043229)	0.001700	1.37	8.72
2	Mitochondrial part (GO:0044429)	0.002309	2.19	13.27
3	Mitochondrial nucleoid (GO:0042645)	0.002648	5.98	35.50
4	Early endosome (GO:0005769)	0.003584	5.52	31.10
5	Nucleoplasm (GO:0005654)	0.004047	1.95	10.75
6	Cytosol (GO:0005829)	0.004356	1.62	8.83
7	Intracellular membrane-bounded organelle (GO:0043231)	0.007203	1.33	6.56

Cellular components analysis of the genes differentially enriched in H3K4me3 in the FDR group. The table shows the GO terms ranked based on *p value* computed from the Fisher’ exact test

### H3K4me3 enrichment on TFAM promoter associates with mRNA reduced expression and mitochondrial DNA stability and transcription in FDR

**Fig. 2:**
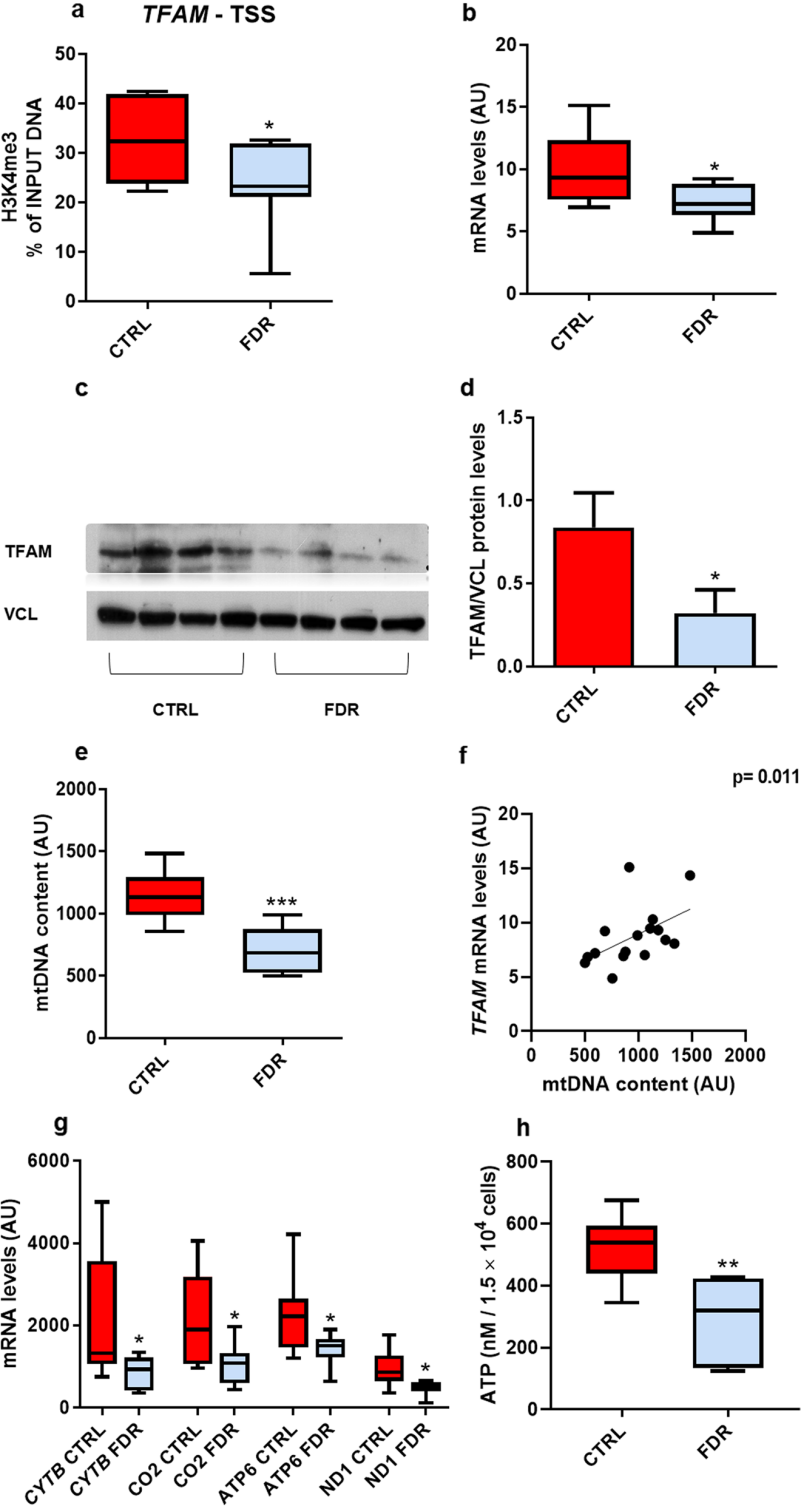
H3K4me3 promoter enrichment associates with reduced TFAM mRNA/protein levels in FDR APCs. **a** H3K4me3 enrichment at the *TFAM* promoter was measured by ChIP-qPCR in subcutaneous APCs from FDR (*n* = 7) and CTRL (*n* = 9). ChIP data were calculated as a percentage relative to the INPUT DNA. **b** *TFAM* mRNA expression levels were measured by qPCR and normalized to *RPL13A* expression in APCs from FDR (*n* = 7) and CTRL (*n* = 9). Values are presented as absolute units (AU). **c**, **d** TFAM protein levels were assessed by western blot in APCs from FDR and CTRL subjects from our study cohort. Vinculin (VCL) served as a protein loading control. The western blot analysis and densitometric measurement of the bands in 4 CTRL and 4 FDR individuals are shown in Figures c and d, respectively. **e** The mtDNA content in subcutaneous APCs from FDR (*n* = 7) and CTRL (*n* = 9) was determined by qPCR. Values are presented as absolute units (AU). **f** Correlation analysis between *TFAM* mRNA levels and mtDNA content in FDR and CTRL APCs. The Spearman’s rank correlation test was used for correlation analysis and assessment of statistical significance (*p *value = *0.011*). **g** *CYTB*, *CO2*, *ATP6*, and *ND1* mRNA expression levels were measured by qPCR and normalized to *TATA-Box binding protein* (*TBP*) expression in APCs from FDR (*n* = 7) and CTRL (*n* = 9). Values are presented as absolute units (AU). **h** ATP concentrations quantified in APCs from FDR (*n* = 7) and CTRL (*n* = 9). **a**, **b**, **e**, **g**, and **h** Data are shown as boxplots (min–max). **a**, **b**, **d**, **e**, **g**, and **h** Statistical significance between the two groups was determined by unpaired Student’s *t*-test (*p value < *0.05*, **p value < 0.01, and ***p value < 0.001 vs CTRL)

We adopted ChIP-qPCR with H3K4me3 antibodies to directly validate the bioinformatic evidence in each member of the study group. To this end, we have first tested the efficacy of ChIP-qPCR for H3K4me3 enrichment using one positive (*Glyceraldehyde 3 Phosphate Dehydrogenase*—TSS) and one negative genomic control region (*Myoglobin exon 2*) (Additional file 1: Fig. S2a). We then analyzed H3K4me3 enrichment in a subgroup of mitochondrial genes, including *TFAM*. Consistent with the ChIp-Seq bioinformatic data, a significant decrease of the H3K4me3 mark was found at the *TFAM* promoter (Fig. 2a) and in all genes analyzed (Additional file 1: Fig. S2b–d) in the FDR compared to CTRL APCs.

Based on this information, we addressed further efforts on *TFAM*, as this gene plays a crucial role in regulating mitochondrial stability and content and is included in the shortlist previously identified by the cellular component merging analysis (Fig. 1c). The decrease of H3K4me3 at *TFAM* promoter in preadipocytes from FDR individuals (*p *value < 0.05; Fig. 2a) was found to be accompanied by a marked reduction in *TFAM* mRNA expression (Fig. 2b; *p *value < 0.05) and protein (Fig. 2c, d; *p *value < 0.05). Furthermore, mitochondrial DNA amount in preadipocytes from FDR subjects was significantly decreased compared to the CTRL (Fig. 2e; *p *value < 0.001) and directly correlated with *TFAM* mRNA expression (Fig. 2f; *p *value = 0.011; Spearman's rank correlation test). Since the C-terminal tail of TFAM is crucial for the activation of promoter-specific transcription of mtDNA [36], we further measured mitochondrial transcription in preadipocytes of the study participants. Interestingly, a reduction in transcription of all four tested mitochondrial-encoded genes [*Cytochrome B* (*CYTB*), *Cytochrome c oxidase II* (*CO2*), *ATP synthase membrane subunit 6* (*ATP6*) and *NADH-ubiquinone oxidoreductase chain 1* (*ND1*)] was measured in FDR preadipocytes (Fig. 2g; *p *value < 0.05). In addition to these alterations, we detected a significant reduction in ATP levels (Fig. 2h) in FDR individuals (*p *value < 0.01).

### Decreased TFAM expression in FDR APCs is associated with reduced histone residues marking transcriptionally active chromatin

**Fig. 3:**
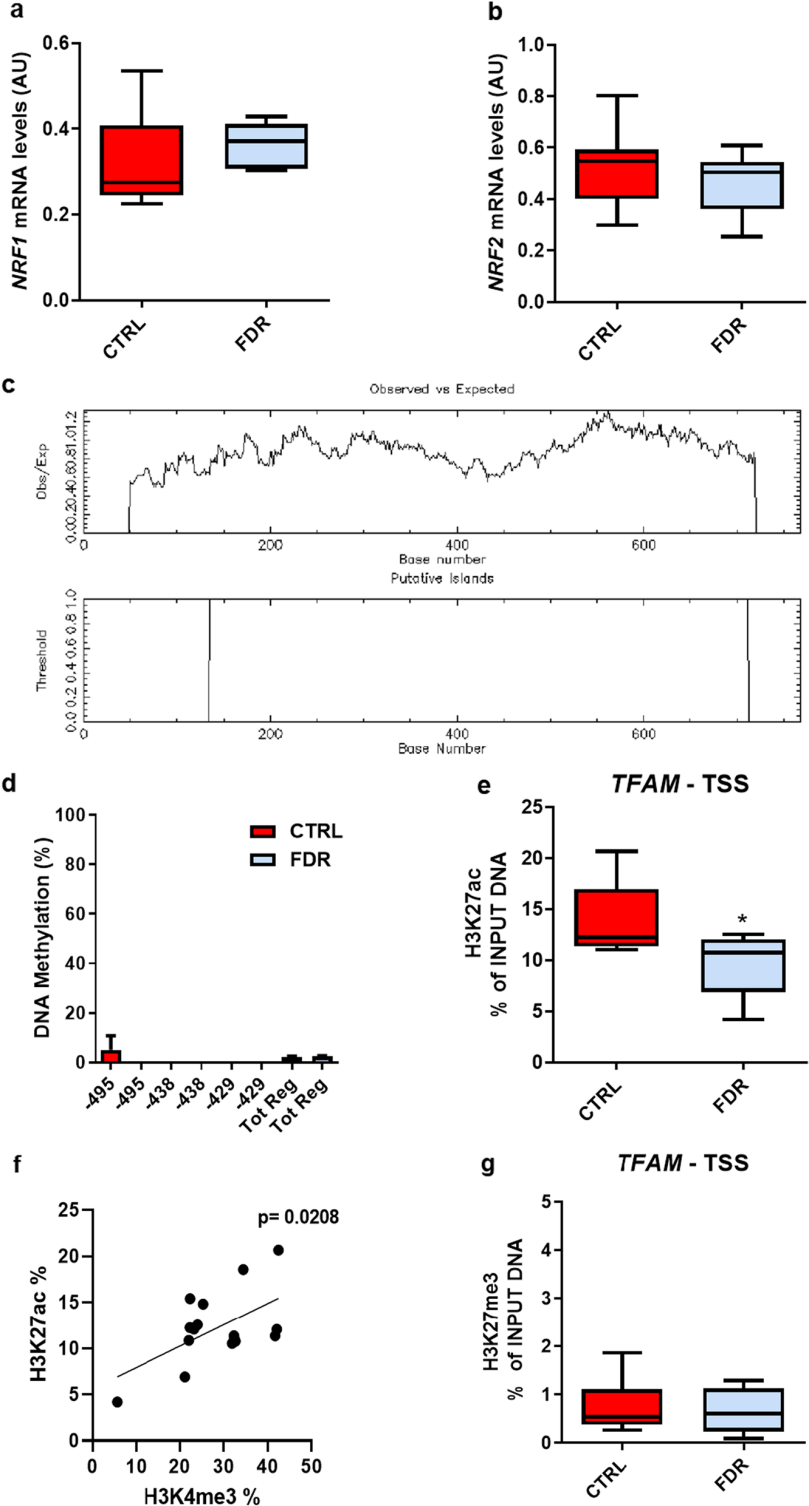
Epigenetic characterization of *TFAM* promoter in CTRL and FDR APCs. **a**, **b** *NRF1* and *NRF2* mRNA expression levels were measured by qPCR and normalized to *RPL13A* expression in APCs from FDR (*n* = 7) and CTRL (*n* = 9). Values are presented as absolute units (AU). **c** A schematic representation of the CpG island (from − 564 to − 20 bp from the TSS) on the *TFAM* promoter identified by the EMBOSS CpGplot software. **d** Quantification of the DNA methylation levels at three specific CpG sites (− 495, − 438, and − 429) and the total region (Tot Reg) in APCs from FDR (blue bars) and CTRL (red bars). **e**, **g** H3K27ac and H3K27me3 enrichment on the *TFAM* promoter measured by ChIP-qPCR in subcutaneous APCs from FDR (*n* = 7) and CTRL (*n* = 9). **f** Correlation analysis between H3K27ac and H3K4me3 enrichment in FDR and CTRL APCs. The Pearson correlation test was used for correlation analysis and statistical significance assessment (*p *value* = 0.0218).* (a, b, e, and g) Data are shown as boxplots (min–max). **a**, **b**, **d**, **e**, and **g** Statistical significance between the two groups was determined by unpaired Student’s *t* test (*p value < 0.05 vs CTRL)

To obtain further insight into the regulation of *TFAM* transcription in APCs, we have examined the *TFAM* upstream transcriptional network, known to be mainly composed of the *Peroxisome proliferator–activated receptor γ coactivator 1α* (*PGC1α*), *Nuclear respiratory factor 1* (*NRF1*), and *Nuclear respiratory factor 2* (*NRF2*) transcription factors. We measured their expression by qPCR and detected no significant difference in mRNA levels of *NRF1* and *NRF2* in the APCs from either FDR or CTRL individuals (Fig. 3a, b), while *PGC1α* was barely detectable in preadipocytes of both subject groups (data not shown). Thus, the differential enrichment of H3K4me3 at *TFAM* promoter was postulated to represent the most proximal change occurring in the FDR subjects and related to the reduced *TFAM* expression in these individuals.

Human studies provide evidence that the expression of the *TFAM* gene is regulated by DNA methylation of its promoter region, and this methylation has been associated with insulin resistance in adolescents [42]. Thus, we further performed DNA methylation analysis at the three differentially methylated CpG sites, which were previously identified and further extended the investigation to the entire CpG island (50 CpG residues) revealed by the EMBOSS CpGplot analysis (Fig. 3c). Bisulphite sequencing of samples from FDR and CTRL individuals showed no different DNA methylation at the − 495, − 438, − 429 residues (Fig. 3d) and at the entire CpG island (Additional file 1: Fig. S3) between these subject groups. Thus, in preadipocytes, the different *TFAM* mRNA expression in FDR and CTRL groups cannot be ascribed to differences in DNA methylation.

We further quantitated H3K27ac and H3K27me3 histone residues, which commonly colocalize with H3K4me3 along the genome and reveal chromatin transcriptional state. To this end, we performed ChIP-qPCR to unveil H3K27me3 and H3K27ac colocalizing with H3K4me3 at *TFAM* promoter in the APCs from FDR and CTRL subjects. Consistent with the reduced *TFAM* expression found in the APCs from FDR individuals, H3K27ac enrichment was significantly decreased at *TFAM*—TSS (Fig. 3e; *p *value < 0.05) and positively correlated with H3K4me3 enrichment (Fig. 3f* p *value = 0.0208*;* Pearson correlation test). At variance, H3K27me3 did not show differences between FDR and CTRL individuals (Fig. 3g). Thus, reduced *TFAM* expression in APCs from individuals who are FDR of T2D is accompanied by decreased enrichment of histone residues that commonly mark actively transcribed chromatin.

### TFAM silencing reduces mtDNA content and mitochondrial-encoded gene transcription in CTRL preadipocytes

**Fig. 4:**
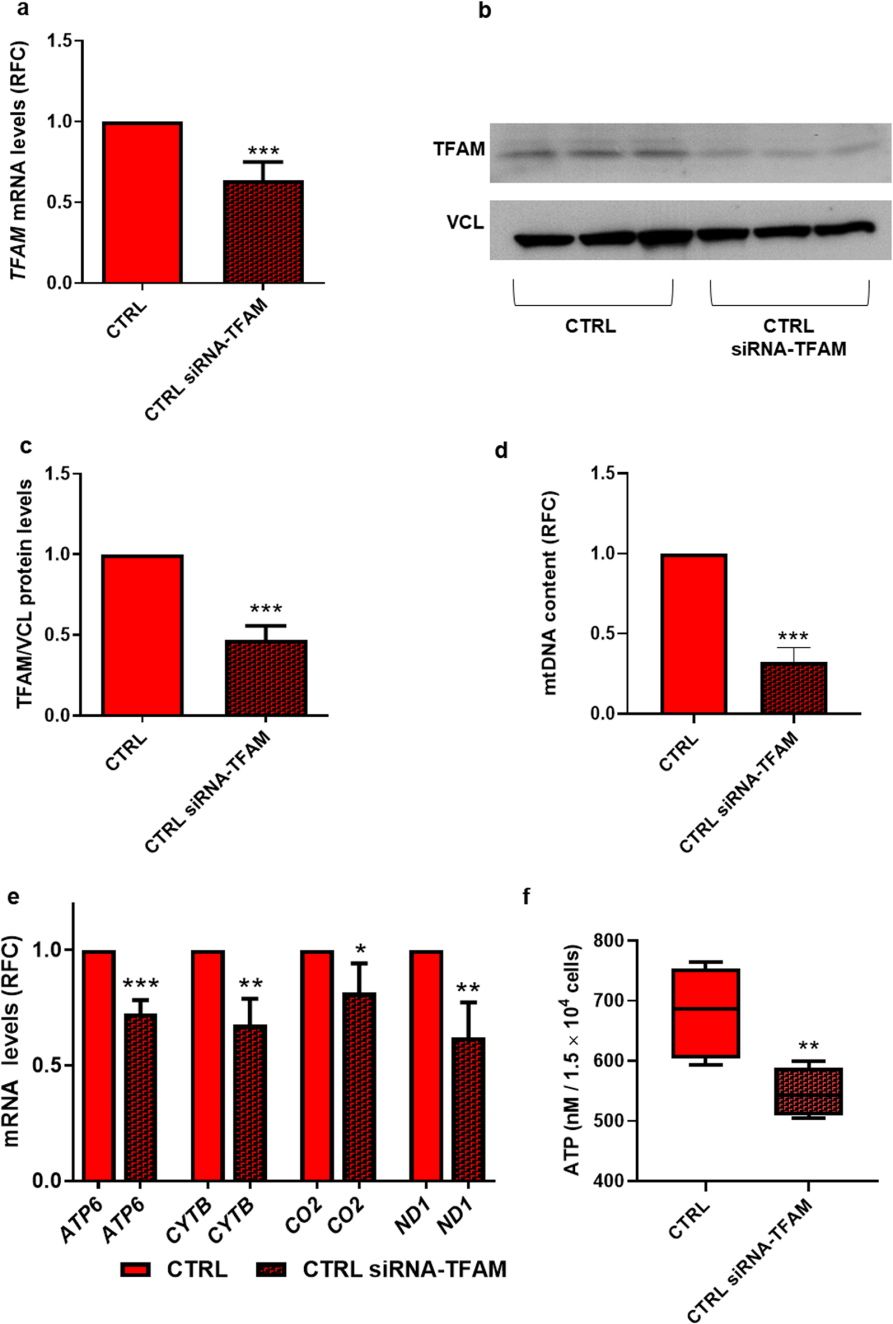
*TFAM* knockdown in CTRL APCs decreases mtDNA content and mitochondrial-encoded gene transcription. **a** *TFAM* mRNA expression levels were measured by qPCR and normalized to *RPL13A* expression in CTRL APCs, and CTRL APCs transfected with *TFAM*-specific siRNA (CTRL siRNA-TFAM) (*n* = 7). Values are presented as relative-fold change (RFC). **b**, **c** TFAM protein levels were assessed by western blot in CTRL and CTRL siRNA-TFAM APCs. Vinculin served as a loading control. Figure **b** shows **a** representative blot (CTRL, *n* = 3; CTRL siRNA-TFAM, *n* = 3); Figure **c** shows the densitometric analysis of proteins bands (CTRL, *n* = 6; CTRL siRNA-TFAM, *n* = 6). **d** The mtDNA content in CTRL and CTRL siRNA-TFAM (n = 4) was measured by qPCR. Values are presented as relative-fold change (RFC). **e** *ATP6*, *CYTB*, *CO2*, and *ND1* mRNA expression levels were measured by qPCR and normalized to *TBP* expression in CTRL and CTRL siRNA-TFAM (*n* = 5). Values are presented as relative-fold change (RFC). **f** ATP concentrations quantified in APCs from CTRL and CTRL siRNA-TFAM (*n* = 5). **a**, **c**, **d**, and **e** Statistical significance was determined by paired Student’s *t* tests (*p value < 0.05, **p value < 0.01, and ***p value < 0.001 vs CTRL). **f** Statistical significance was determined by unpaired Student's *t* tests (**p value < 0.01 vs CTRL)

To examine the functional role of TFAM, we adopted siRNA technology and silenced *TFAM* expression in CTRL APCs. As shown in Fig. 4a, transfection of the preadipocytes with *TFAM*-specific siRNA downregulated the endogenous *TFAM* mRNA expression by ~ 50% (*p *value < 0.001) compared to the control cells (transfected with scramble siRNA). Based on western blot analysis, TFAM protein levels were similarly decreased in the siRNA-treated cells (Fig. 4b, c,* p *value < 0.001). Reminiscent of the FDR individuals who feature reduced *TFAM* expression (Fig. 2e, f), siRNA silencing of *TFAM* in the preadipocytes caused a significant reduction in the content of mtDNA (*p *value < 0.001; Fig. 4d) and in the expression of mitochondrial-encoded genes *ATP6* (p value < 0.001), *CYTB* (*p *value < 0.01), *CO2* (*p *value < 0.05), and *ND1* (*p *value < 0.01) (Fig. 4e). Silencing of *TFAM* in CTRL APCs also resulted in a significant decrease in cellular ATP levels (*p *value < 0.01) (Fig. 4f).

These novel findings confirmed the fundamental role of *TFAM* in regulating mtDNA content, mitochondrial-encoded gene transcription, and ATP supply. This strengthened the possibility that the epigenetic dysregulation of *TFAM* expression found in individuals who are FDR is relevant to the APCs dysfunction in these subjects.

### Impaired adipocyte differentiation ability in FDR APCs is closely associated with mitochondrial alterations

**Fig. 5:**
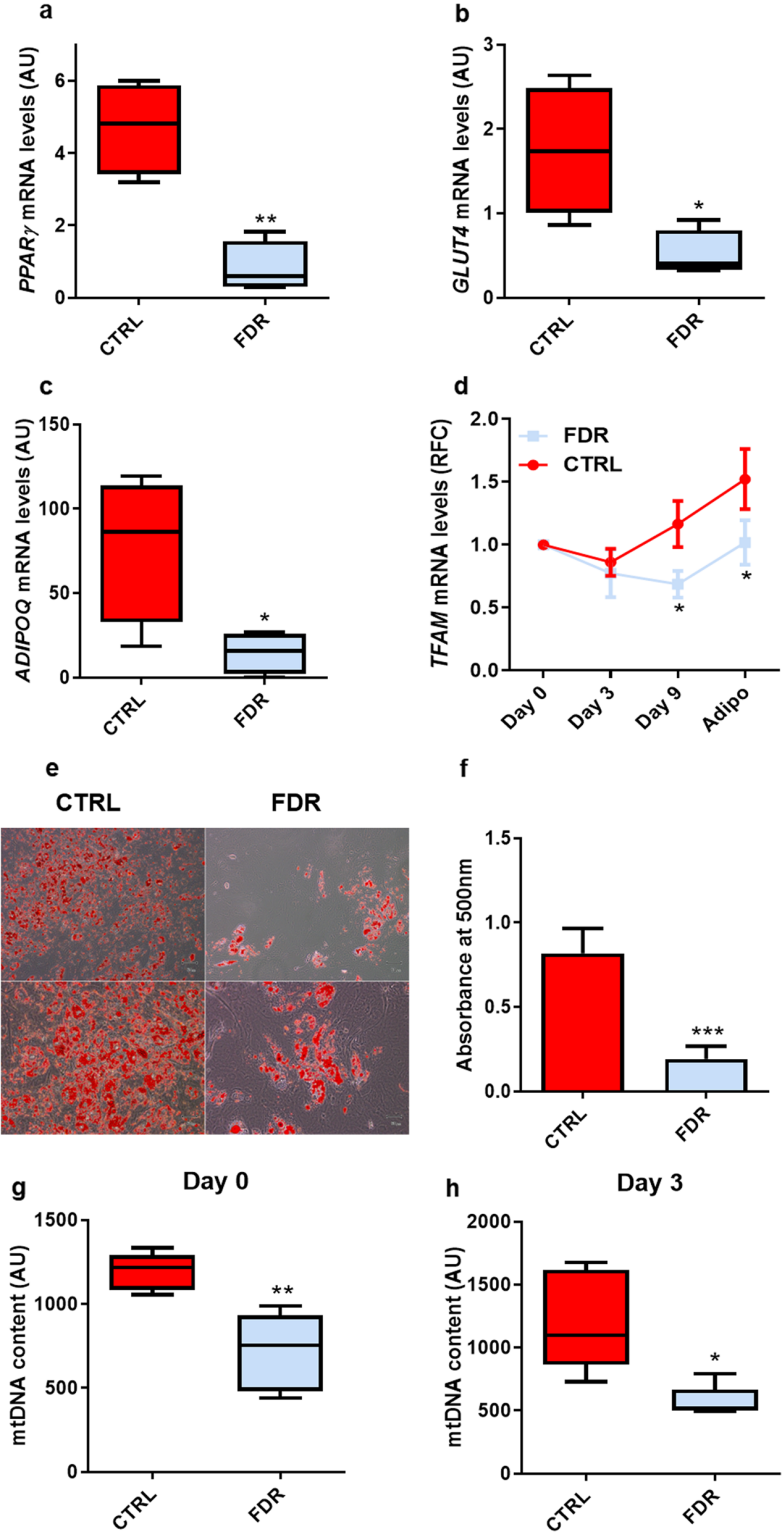

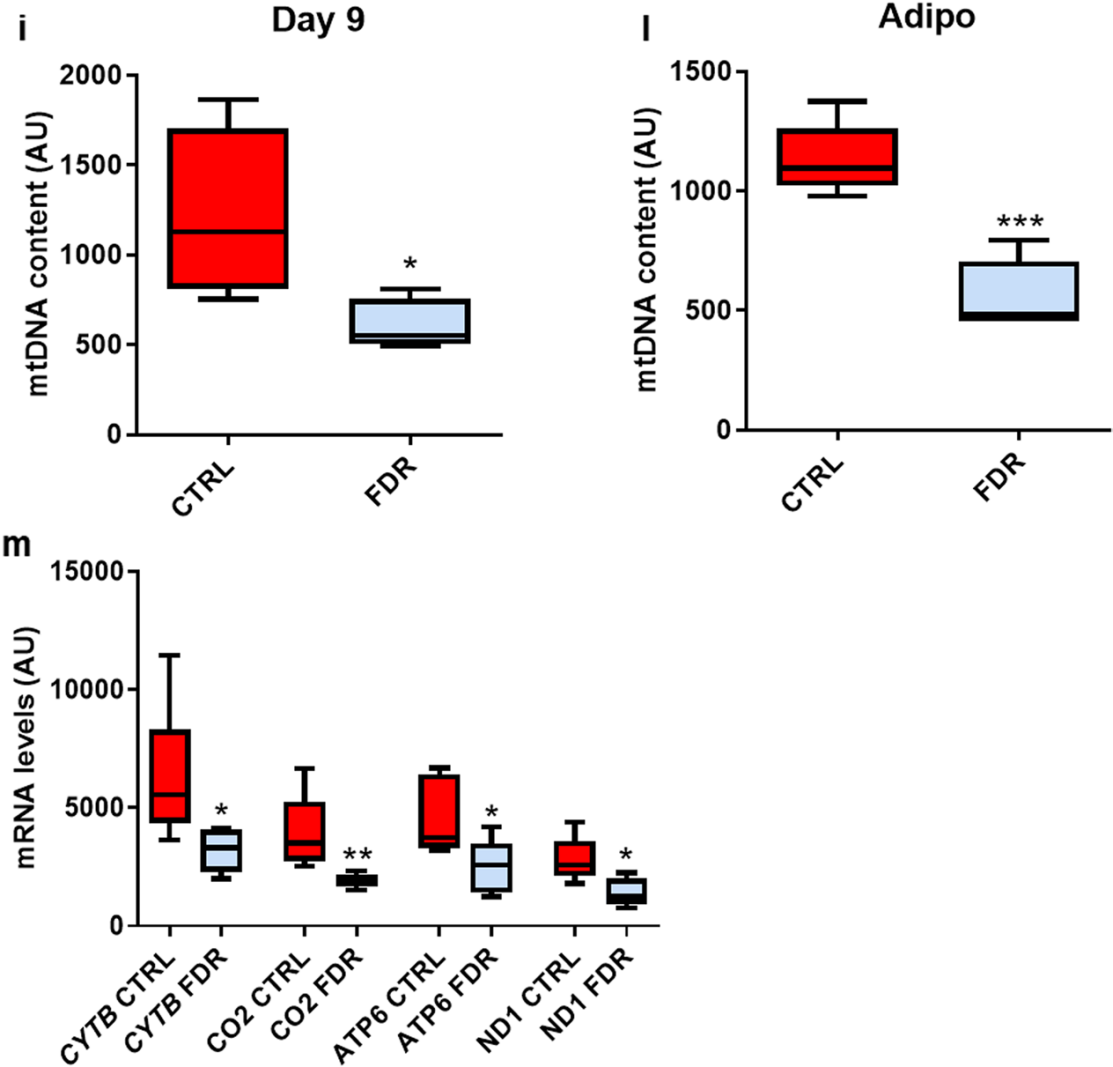
mtDNA content and impaired adipogenic differentiation in FDR APCs. **a**–**c** At the end of adipocyte differentiation, the expression of adipogenic marker genes *PPARγ*, *GLUT4*, and *ADIPOQ* normalized to *RPL13A* expression was determined by qPCR in FDR (*n* = 4) and CTRL (*n* = 4) APCs. Values are presented as absolute units (AU). **d** *TFAM* mRNA expression levels were measured by qPCR in APCs from FDR (*n* = 4) and CTRL (*n* = 4) on days 0, 3, 9, and 15 of adipocyte differentiation. Values are presented as relative-fold change (RFC) and were normalized to *RPL13A* expression. **e** Oil Red O staining was used for assessing the differentiation degree. Representative microphotographs showing lipid accumulation of CTRL (left) and FDR (right) APCs on day 15 of the differentiation process. Upper panel at 10 × magnification; bottom panel at 20×magnification. Scale bar 50 μm. **f** Quantification of Oil Red O staining (CTRL, *n* = 5; FDR, *n* = 5). (**g**–**l**) The mtDNA content was measured by qPCR in APCs from FDR (*n* = 5) and CTRL (*n* = 5) on days 0, 3, 9, and at the end of adipocyte differentiation. Values are presented as absolute units (AU). **m** *CYTB*, *CO2*, *ATP6*, and *ND1* mRNA expression levels were measured by qPCR and normalized to *TBP* expression in APCs from FDR (*n* = 6) and CTRL (*n* = 6). Values are presented as absolute units (AU). (**a**–**c**, and **g**–**m**) Data are shown as boxplots (min–max). (**a**–**d**, **f**, and **i**–**m**) Statistical significance between the two groups was determined by unpaired Student’s *t*-test (*p value < *0.05*, **p value < 0.01, and ***p value < 0.001 vs CTRL). **h** Statistical significance between the two groups was determined by unpaired Mann–Whitney (*p value < 0.05 vs CTRL)

FDR individuals exhibit SAT dysfunction associated with adipocyte hypertrophy and defective adipocyte precursor cell recruitment into the adipogenic program [9]. To explore whether the mitochondrial alterations found in individuals who are FDR impact human adipogenesis, we first compared, in FDR and CTRL subjects, the mRNA level changes of several adipogenesis marker genes through *in vitro* adipogenesis. It was shown that the cells from the FDR subjects exhibited significantly lower levels of *Peroxisome proliferator-activated receptor γ* (*PPARγ*), *Glucose transporter type 4* (*GLUT4*) and *Adiponectin* (*ADIPOQ*) compared to those from CTRL individuals (Fig. 5a* p *value < 0.01; 5b, *p *value < 0.05; 5c, *p *value < 0.05). Oil Red O staining of these cells, shown in Fig. 5e at two different magnifications, also revealed that CTRL adipocytes feature > 3.5-fold increased capability to accumulate lipid droplets compared to FDR adipocytes (Fig. 5f* p *value < 0.001). Interestingly, *TFAM* mRNA levels across adipocyte differentiation were found to steadily increase in CTRL APCs (Fig. 5d; significant at days 9 and 15 at the *p value* < *0.05* levels compared to day 0). Surprisingly though, *TFAM* mRNA did not increase in the FDR APCs. In addition, mtDNA content during adipogenesis was also lower in FDR compared to CTRL individuals (Fig. 5g, *p* value < 0.01; 5 h, *p* value < 0.05; 5i, *p* value < 0.05; 5l, *p* value < 0.001), as well as the mitochondrial-encoded gene transcription CYTB (*p* value < 0.05), CO_2_ (*p* value < 0.01), ATP6 (*p* value < 0.05), ND1 (*p* value < 0.05) (Fig. 5m).

### *TFAM* silencing impairs adipocyte differentiation and reduces mtDNA and mitochondrial transcription in mature adipocytes

**Fig. 6:**
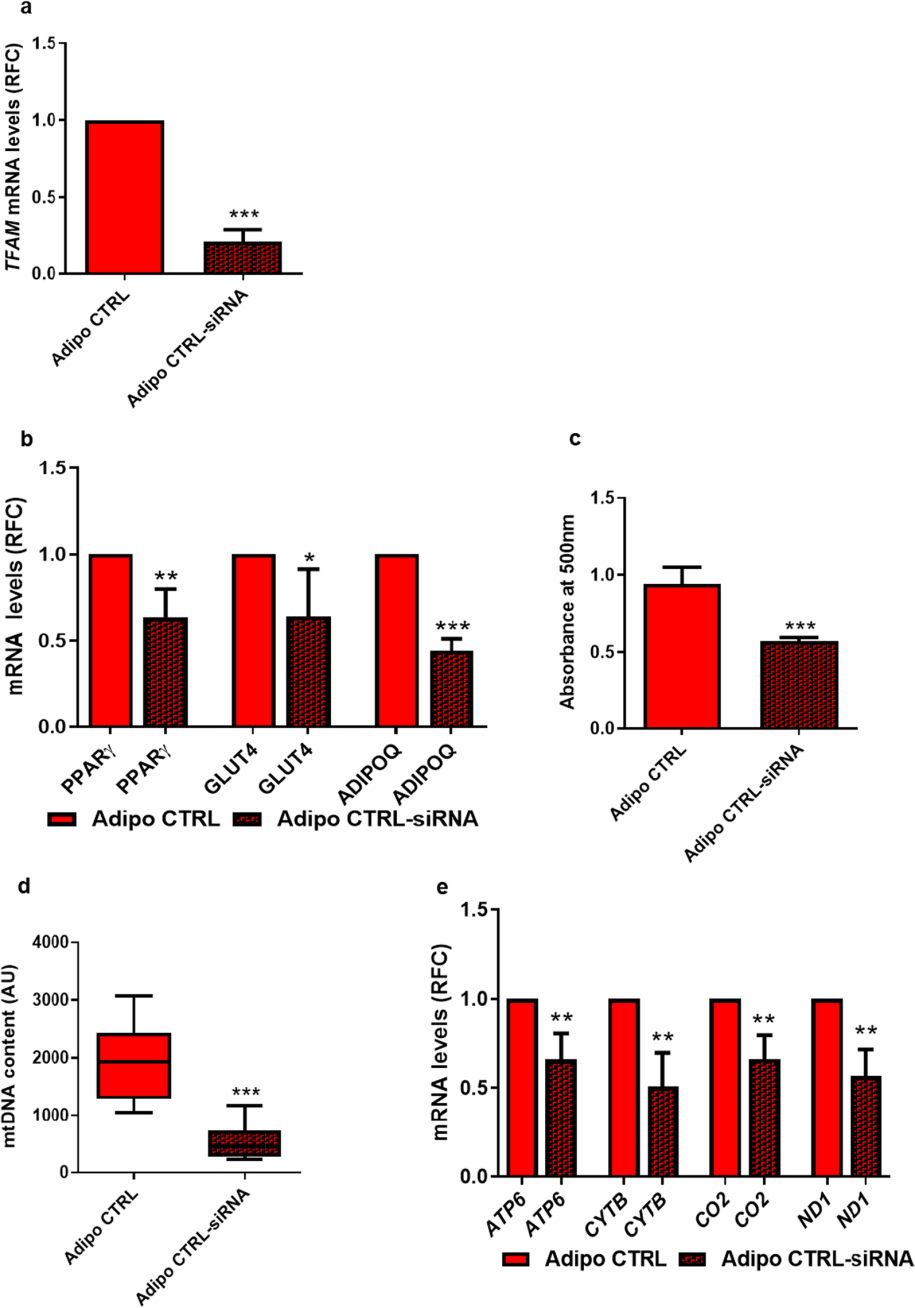
*TFAM* knockdown causes reduction in adipogenic potential of CTRL APCs. **a** On day 15 of the differentiation process, *TFAM* mRNA expression levels were measured by qPCR and normalized to *RPL13A* expression in CTRL adipocytes (Adipo CTRL) and CTRL *TFAM*-silenced adipocytes (Adipo CTRL-siRNA) (*n* = 5). Values are presented as relative-fold change (RFC). **b** The expression of adipogenic marker genes *PPARγ*, *GLUT4*, and *ADIPOQ* mRNA levels was measured by qPCR and normalized to *RPL13A* expression in Adipo CTRL and Adipo CTRL-siRNA (*n* = 5). Values are presented as relative-fold change (RFC). **c** Quantification of Oil Red O staining (*n* = 6). **d** The mtDNA content was measured by qPCR in Adipo CTRL and Adipo CTRL-siRNA (*n* = 6). Values are presented as absolute units (AU), and their distribution within each group is represented by box plots. **e** *ATP6*, *CYTB*, *CO2*, and *ND1* mRNA expression levels were measured by qPCR and normalized to *TBP* expression in Adipo CTRL and Adipo CTRL-siRNA (*n* = 5). Values are presented as relative-fold change (RFC). **a**, **b**, **d**, and **e** Statistical significance was determined by paired Student’s *t*-test (*p value < 0.05, **p value < 0.01, and ***p value < 0.001 vs Adipo CTRL). **c** Statistical significance between the two groups was determined by unpaired Mann–Whitney (*p value < 0.001 vs Adipo CTRL)

Mitochondrial function and content are critical for adipogenesis, and we now report a major *TFAM* engagement in regulating mtDNA content and mitochondrial gene transcription in APCs. Therefore, we directly addressed the hypothesis that *TFAM* knockdown affects adipocyte differentiation of APCs by silencing *TFAM* through the entire *in vitro* adipogenic course (Fig. 6a). Transcriptional *TFAM* silencing (Fig. 6a,* p *value < 0.001) significantly reduces the mRNA levels of *PPARγ* (*p *value < 0.01), *GLUT4* (*p *value < 0.05), and *ADIPOQ* (*p *value < 0.001) genes (Fig. 6b), accompanied by decreased capability in lipid droplet accumulation, as shown by the Oil Red O staining quantification (Fig. 6c,* p value* < *0.001*). Therefore, when *TFAM* expression is reduced, as it occurs in subjects who are FDR, the APCs of the control individuals also exhibit impairment in their capability to completely differentiate. Control experiments further revealed that mtDNA content (Fig. 6d; *p *value < 0.001), as well as the expression of the mitochondrial-encoded genes *ATP6* (*p *value < 0.01), CYTB (*p *value < 0.01), CO_2_ (*p *value < 0.01), and *ND1* (*p *value < 0.01) (Fig. 6e) were decreased in the *TFAM*-silenced mature adipocytes. Thus, *TFAM* expression is essential for mitochondrial content and function and critical for the accomplishment of the complete APCs differentiation program.

### *TFAM* contributes to the acquisition of premature senescence in APCs from FDR individuals

**Fig. 7:**
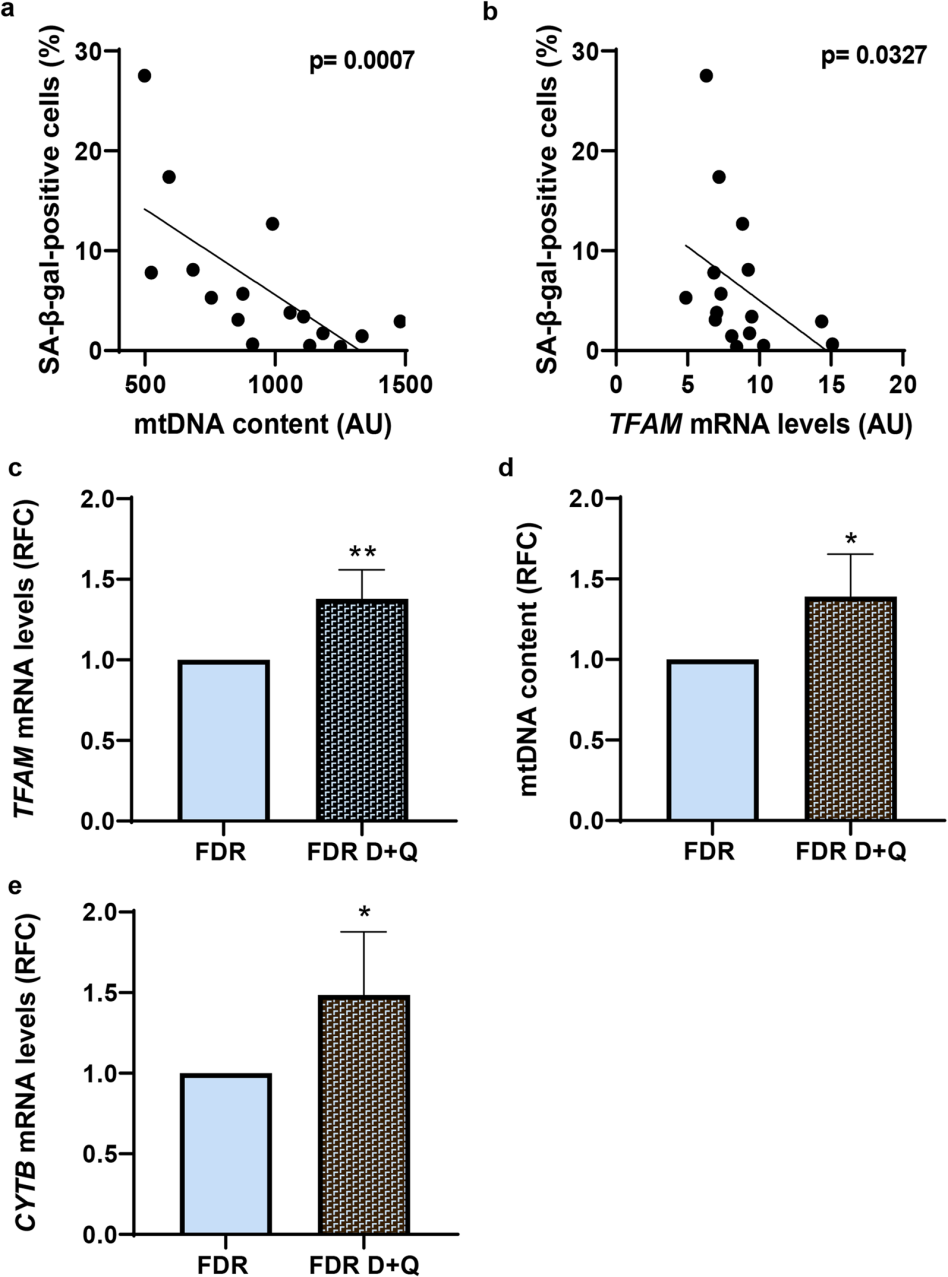
Mitochondrial alterations and the development of premature senescence in FDR APCs. **a**, **b** Correlation analysis between the percentage of SA-β-gal-positive cells and mtDNA content (*p *value = 0.0007), and *TFAM* mRNA levels (*p *value = 0.0327) in FDR and CTRL APCs. The Spearman's rank correlation test was used for correlation analysis and statistical significance assessment. **c**–**e** *TFAM*, and *CYTB* mRNA expression levels were measured by qPCR and normalized to *RPL13A* and *TBP* expression, respectively, in FDR APCs treated with a combination of Dasatinib (D) and Quercetin (Q) (FDR D + Q) (*n* = 6). Values are presented as relative-fold change (RFC). **d** mtDNA content in the APCs from FDR and FDR D + Q (*n* = 6) was measured by qPCR. Values are presented as relative-fold change (RFC). **c**–**e**. Statistical significance was determined by paired Student's t-test (*p value < 0.05, and **p value < 0.01 vs FDR)

We have recently reported that FDR individuals feature subcutaneous preadipocytes early senescence, which contributes to their inability to fully differentiate into mature adipocytes [43]. Thus, we have further addressed the relation between mitochondrial alterations and the development of FDR APCs senescence. Interestingly, a negative correlation was observed between senescence-associated β-galactosidase (SA-β-gal) positive cells and mtDNA amount (Fig. 7a,* p *value = 0.0007; Spearman’s rank correlation test). Indeed, SA-β-gal positive cells negatively correlated with *TFAM* mRNA expression (Fig. 7b,* p value* = *0.0327*; Spearman’s rank correlation test). Furthermore, treatment of FDR APCs with a senolytic combinations of Dasatinib and Quercetin that selectively eliminates senescent preadipocytes, as we previously published [43], was paralleled by enhanced: *(i) TFAM* mRNA expression (Fig. 7c,* pvalue* < *0.01*); (ii) mtDNA content (Fig. 7d,* p *value < 0.05); and *iii)* mitochondrial-encoded gene transcription (Fig. 7e,* p *value < 0.05). Senolytic combinations were further able to improve adipocyte differentiation in FDR APCs (data not shown), as we already reported [43].

**Fig. 8:**
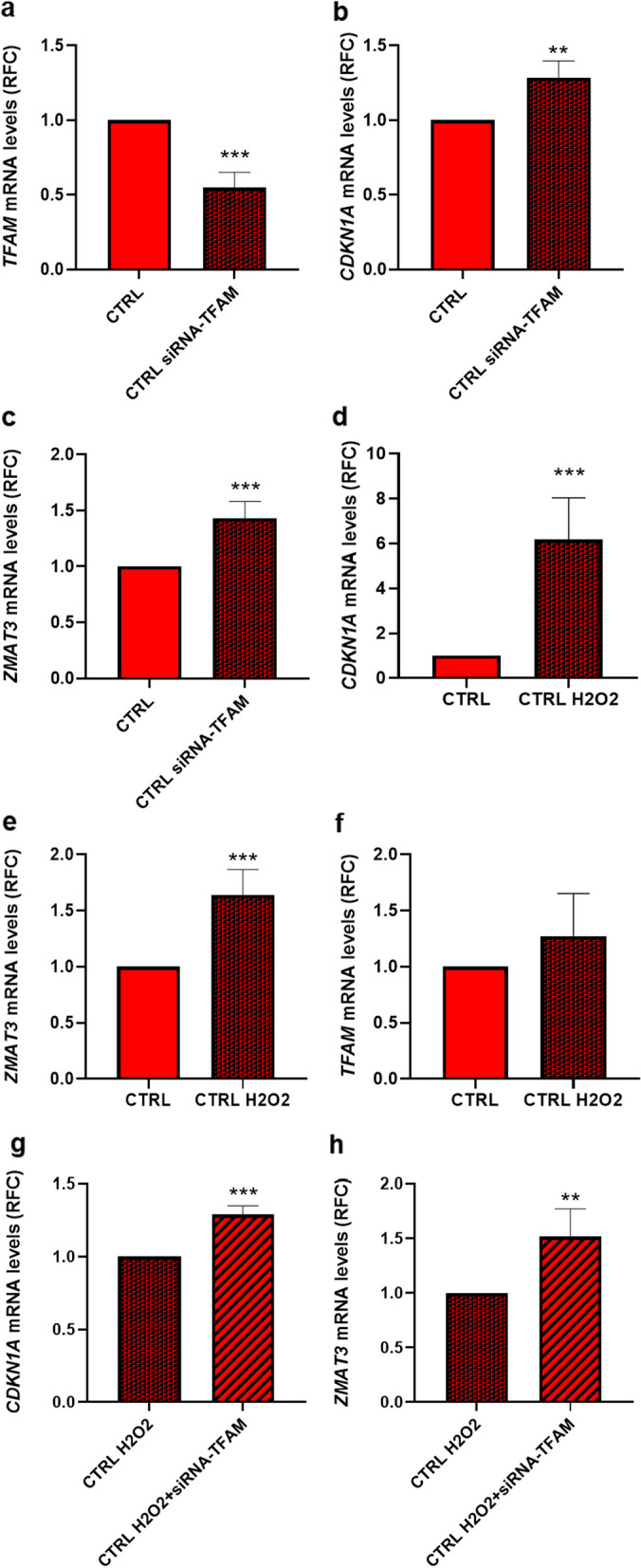
The effect of *TFAM* expression in induction of a premature senescent phenotype in FDR APCs. **a**–**c** *TFAM*, *CDKN1A*, and *ZMAT3* mRNA expression levels were measured by qPCR and normalized to *RPL13A* expression in CTRL *TFAM*-silenced APCs (CTRL siRNA-TFAM) (*n* = 6). Values are presented as relative-fold change (RFC). Statistical significance between the two groups was determined by paired Student’s *t*-test (**p value < 0.01, and ***p value < 0.001 vs CTRL). **d**–**f** *CDKN1A*, *ZMAT3*, and *TFAM* mRNA expression levels were measured by qPCR and normalized to *RPL13A* expression in CTRL APCs exposed to (CTRL H2O2) or not to H_2_O_2_ (*n* = 6). Values are presented as relative-fold change (RFC). Statistical significance between the two groups was determined by paired Student’s *t*-test (***p value < 0.001 vs CTRL). (**g**, **h**) *CDKN1A*, and *ZMAT3* mRNA expression levels were measured by qPCR and normalized to *RPL13A* expression in CTRL APCs exposed to H_2_O_2_ (CTRL H2O2) and silenced for *TFAM* gene (CTRL H2O2 siRNA-TFAM) (*n* = 6). Values are presented as relative-fold change (RFC). Statistical significance between the two groups was determined by paired Student’s *t*-test (***p value < 0.001, and **p value < 0.01 vs CTRL H2O2)

Thus, to investigate whether a change in *TFAM* mRNA has direct effects on senescence, we assessed the expression of senescence marker genes in *TFAM*-silenced CTRL preadipocytes. *TFAM* silencing (Fig. 8a,* p *value < 0.001) caused a slight but significant increase in *Cyclin-dependent kinase inhibitor 1A* (*CDKN1A*) mRNA level (Fig. 8b,* p *value < 0.01) as well as in the expression of the senescence marker *Zinc finger matrin-type 3* (*ZMAT3*) gene (Fig. 8c,* p *value < 0.001). Whether premature senescence in APCs causes downstream changes in *TFAM* expression was also tested by exposing the cells from CTRL subjects to the senescence inducer hydrogen peroxide (H_2_O_2_). As anticipated based on our previous findings [43], upon H_2_O_2_ treatment of CTRL APCs, upregulation of the CDKN1A (Fig. 8d, *p* value < 0.001) and ZMAT3 (Fig. 8e, *p* value < 0.001) occurred. These effects were not paralleled by alterations in TFAM expression though (Fig. 8f). In addition, TFAM-siRNA silencing amplified the effect of H_2_O_2_ on both CDKN1A (*p* value < 0.001) (Fig. 8g) and ZMAT3 expression (*p* value < 0.01) (Fig. 8h), strengthening the hypothesis that reduced TFAM mRNA impacts premature APCs senescence.

## Discussion

Adipose tissue hypertrophy and dysfunction have long been recognized as major determinants of metabolic complications in both obese and non-obese individuals who are at excess risk of T2D [4–10]. Here, we have explored the molecular mechanisms determining adipose tissue dysfunction in healthy non-obese subjects who are FDR of T2D. These individuals represent a unique study population, which features a high risk of T2D [15–17]. They exhibit SAT hypertrophy and dysfunction similar to those of T2D enabling the analysis of mechanisms responsible for these adipose tissue abnormalities without obesity confounders. Previous reports have indicated that, in these FDR, subcutaneous adipocyte hypertrophy is a consequence of impaired APCs recruitment in the adipogenic program and not of APCs lack [21–23]. However, the detailed molecular mechanisms responsible for defective APCs lineage determination in FDR remain incompletely understood.

APCs determination to the adipocyte lineage is epigenetically regulated [31, 32], and epigenetic mechanisms also provide an important contribution to the missing heritability of T2D [29, 44]. Recently, we have shown that SAT hypertrophy in FDR is contributed by changes in DNA methylation at several loci and by abnormalities in the expression of different microRNAs in the SAT, some of which also reflect similar changes in peripheral blood leukocytes [27, 28]. Here, we have further explored the presence of histone modification profiles in APCs from FDR of T2D, contributing to APCs differentiation and fat tissue hypertrophy in these individuals. We have adopted a genome-wide approach and focused on H3K4me3, which is strictly associated with transcriptional activity and cellular identity, regulating essential biological processes and stem cell biology [35]. In addition, current evidence indicates that the H3K4me3 profile is enriched at the promoter of key adipogenic genes, and its co-localization with other specific histone marks discriminates the transcriptional states (repressed, poised, or activated) of a number of genes and the stemness degree of APCs [45].

We generated and comparatively analyzed the whole-genome H3K4me3 profiles in both FDR and CTRL APCs. As expected, H3K4me3 sharper peaks exhibited a characteristic localization across 500–1500 base pairs, in most cases in the close proximity of annotated TSS [35, 45, 46]. Most of the H3K4me3 differentially enriched regions in the genome of APCs from FDR individuals overlapped with mitochondria-related genes (encoded by the nuclear genome), as further validated by ChIP-qPCR analysis in each individual subject of the study population. These findings were intriguing, as previous reports indicated that reduced mitochondrial activity occurs in obesity, T2D and metabolic syndrome [1–3]. In particular, studies in BMI-discordant monozygotic twins revealed that the downregulation of mitochondrial pathways leads to SAT hypertrophy, increased liver fat accumulation, and deteriorated metabolic profile in the metabolically unhealthy twin, consistent with the contribution of epigenetic mechanisms to these abnormalities [47–49].

The overlapping genes belonging to the three most enriched GO terms return a shortlist of four shared genes, and we addressed further efforts on *TFAM*, as this gene plays an essential role in regulating mitochondrial DNA stability and content. Besides *TFAM*, other mitochondrial-related genes, encoded by the nuclear genome, resulted in being differently enriched in H3K4me3 in FDR individuals (Table S2). Therefore, we cannot exclude that other aspects of mitochondrial function can also be impaired by other genes listed in our dataset beyond the alterations directly caused by TFAM. Although this aspect will be a matter of our future investigations, it represents a limitation of the present study.

In FDR preadipocytes, the *TFAM* gene showed a significant decrease of the H3K4me3 mark at its promoter, accompanied by reduced mRNA expression. TFAM, a member of the high-mobility-group family of genes, is the first mitochondrial transcription factor to be identified in mammalian cells [50, 51]. In the mitochondria, this transcriptional activator is essential for mtDNA expression and maintenance [52]. *TFAM* upstream transcriptional network (*NRF1*, *NRF2*, *PGC1α*) was found unchanged in FDR individuals leading us to suggest that the reduced enrichment of H3K4me3 found in FDR is the most proximal abnormality in its pathway.

Typically, epigenetic regulatory mechanisms coordinately act in reprogramming gene expression. Thus, to investigate *TFAM* chromatin transcriptional state in greater detail, we further analyzed DNA methylation. Indeed, evidence from human studies indicates that *TFAM* gene transcription is regulated by DNA methylation of its promoter [53], and this correlates with the onset of insulin resistance in adolescents [42]. In this study, bisulphite sequencing did not reveal any difference in FDR and CTRL APCs as the *TFAM* promoter was almost demethylated in both FDR and CTRL subjects, consistent with the high expression rate observed in the human preadipocytes. We suggest that the apparent discrepancy with the work by Gemma and coworkers [42] is attributable to the different cell type analyzed in our study, as not rarely epigenetic modifications are tissue specific.

Furthermore, we add novel details to the histone regulation of the *TFAM* gene in humans and confirm preliminary observations obtained in a study on pancreatic islets of the intrauterine growth retardation rat model [54]. The co-localization of H3K4me3 with other histone marks directly unveil chromatin transcriptional state, so we further analyzed the H3K27ac and H3K27me3 marks [45]. While the H3K27ac enrichment was found significantly decreased at the *TFAM—*TSS in FDR preadipocytes, the enrichment for H3K27me3 did not show difference between FDR and CTRL APCs and was almost lacking at *TFAM* promoter in both groups. In embryonic stem cells, it is common to observe the presence of both active H3K4me3 and repressive H3K27me3 marks on gene promoters. The ‘‘bivalent domains’’ maintain pluripotency by silencing developmental genes expression [45, 55], enabling the loaded RNA polymerase II to be paused within the bivalent domain [56]. During the progression of lineage specification, bivalent domains undergo resolution and transition into monovalent marks [57]. This transition involves the loss of the repressive H3K27me3 mark and an expansion of the active H3K4me3 on activated genes [40, 45]. Thus, our *TFAM* promoter data indicate that H3K27me3 is resolved both in FDR and in CTRL preadipocytes, while the gene is not maintained in a poised status. The data further show that the reduced enrichment of both H3K4me3 and H3K27ac at the promoter region enables the reduced *TFAM* transcription rate observed in APCs from individuals who are FDR.

*In vivo* studies in knockout animals and other silencing approaches also showed a direct role of *TFAM* in regulating whole-body energy homeostasis. Selective ablation of *TFAM* in mouse WAT causes severe mitochondrial alterations, accompanied by subcutaneous adipocyte loss and WAT inflammation. Lipodystrophy was further exacerbated when these mice were fed a high-fat diet, as the animals developed whole-body insulin-resistance, hepatosteatosis, and cardiac dysfunction [58]. *TFAM* knockout also impaired mtDNA content and mitochondrial transcription in 3T3-L1 preadipocytes [59]. Silenced adipocytes exhibited reduced insulin sensitivity, *Glut4* mRNA expression, and adiponectin secretion [59]. Albeit with much greater severity, the consequences of *TFAM* loss in these models recapitulate those determined by the impaired expression in the FDR preadipocytes. The reduced mtDNA and transcription of mitochondrial-encoded genes found in these subjects are also consistent with previous findings showing TFAM tail region essential for precise DNA recognition, mitochondrial transcriptional activation and priming DNA synthesis during mtDNA replication [36]. Indeed, in zebrafish and mice embryos, the absence of TFAM results in impaired transcription of mitochondrial DNA, an inability to maintain mtDNA integrity, and suffer bioenergetic failure, which ultimately leads to embryonic death [52, 60]. Thus, our findings in the adipose precursor cells from FDR confirm the pivotal role of TFAM protein in regulating mtDNA content and mitochondrial transcription in humans.

Adipocyte precursor differentiation into mature adipocytes is precisely orchestrated by sequential changes in a number of transcription factors, including C/EBP members and PPARγ, which, in turn, drive the expression of a further downstream gene network [4, 61]. In white adipocytes, mitochondria are crucial due to their involvement in adipogenesis, lipid metabolism, and ATP energy production [62]. Several studies indicate that mitochondrial biogenesis and adipogenesis are indeed intertwined since PPARγ, C/EBPα, and PGC1α are indispensable to both processes [61]. The induction of adipocyte differentiation is accompanied by an increase in mitochondrial mass and an enhancement in mitochondrial gene expression, which are essential to adequately meet the increased energy requirements during the differentiation program [63]. Increased ATP demand during adipogenesis supports the metabolic changes and highly energy-consuming lipogenic processes required for proper adipocyte differentiation and function [61, 62]. Inhibitors of mitochondrial oxidative phosphorylation and mitochondrial protein synthesis determine abnormal lipid accumulation and impaired adipogenesis [64]. Reduced ATP content, caused by the downregulation of the *TFAM* expression in FDR APCs, renders the cells incapable of meeting the energy requirement to address the differentiation program. As a consequence, the observed impairment in adipogenesis among FDR subjects could also be attributed to the observed decline in ATP levels, and this represents an essential contribution of our study to the research field. Based on this, we propose that the mitochondrial dysfunction found in the preadipocytes also renders mature adipose tissue dysfunctional in subjects who are FDR, impairing normal hyperplastic response and switching on compensatory hypertrophy of the resident adipocytes and subsequent inflammation. Also, we have shown that *TFAM* silencing inhibits adipocyte differentiation of APCs from CTRL donors, as demonstrated by decreased lipid droplet accumulation and reduced adipocyte marker gene expression. Additionally, *TFAM* silencing leads to a reduction in the amount of mtDNA and impairs the mitochondrial-encoded gene transcription in the silenced mature adipocytes. Thus, the early epigenetic abnormality in *TFAM* regulation established in precursor cells later affects adipose tissue function in FDR individuals. Of note, this abnormality seems independent of obesity and precedes T2D debut as the FDR individuals we have investigated were healthy non-obese subjects.

We recently reported that the restrained adipogenesis in both T2D/FDR and in obese subjects occurs in APCs which feature premature senescence [19, 65], suggesting that similar cellular senescence and/or other mechanisms responsible for cell aging are causative determinants in these individuals. We and others have also shown that DNA methylation is a key regulatory switch through which senescence genes impair APCs function in subcutaneous fat [43, 66]. Mitochondrial biogenesis decline also represents a pillar of molecular aging and cellular senescence [67–69]. Even more recently, different studies have implicated decreased *TFAM* mRNA expression in senescence [68, 70, 71]. In peripheral blood T cells, impaired *TFAM* expression causes premature senescence and multimorbidity (including metabolic, cognitive and cardiovascular disorders) [71]. Consistent with these findings, we found that both *TFAM* mRNA and mtDNA show a negative correlation with the percentage of senescence-positive APCs. *TFAM*-siRNA directly affects the expression of senescence marker genes, as already shown in other tissues [71, 72]. In addition, we show that H_2_O_2_-induced senescence, which has no effect on *TFAM* mRNA expression, is further exacerbated by *TFAM* silencing, supporting the implication of mitochondrial dysfunction in the appearance of premature senescence in APCs from FDR individuals. In these cells, the dysfunctional mitochondria due to *TFAM* deficiency may act as senescence accelerators. The reduction of senescent cell burden by the combination of Dasatinib and Quercetin consistently confirms that dysfunctional mitochondria and *TFAM* deficiency represent a signature of FDR APCs senescence. The impact that senolytics could have on the mitochondrial alterations predisposing to senescence and the associated metabolic complications is not yet fully understood. Senolytics treatment of FDR APCs leads to an increase in *TFAM* expression, increase in mtDNA content and increase in mitochondrial transcription, also improving APCs ability to differentiate into mature adipocytes and reducing tissue hypertrophy, as we previously published [43]. In this scenario, the clearance of senescent cells burden could represent a feasible *in vivo* therapeutic opportunity to overcome mitochondrial alterations and premature senescence in FDR APCs.

The Unitary Theory of Fundamental Aging Mechanisms grows on the geroscience hypothesis by proposing that interventions addressing any fundamental aging mechanism could potentially have broader effects on multiple aspects of the aging process [69]. If this postulate holds, in the future, *TFAM* will represent an interesting target for investigative strategies of both pharmacological and lifestyle intervention.

## Conclusion

In this study, we report a genome-wide histone profile of APCs from healthy individuals with T2D family risk, making it possible to identify new mechanistic insight accounting for SAT dysfunction. The decline of mitochondrial mass and transcription, resulting in a reduced ATP supply, represent unexplored traits that characterize the altered APCs functionality of healthy subjects at increased T2D risk. We identify APCs mitochondrial alterations as a critical player in SAT dysfunction and shed light on the TFAM role in determining these events, enabling the future identification of targeted intervention approaches. Targeting mitochondrial mass and function by senolytic drugs could generate opportunities to overcome the restricted subcutaneous adipogenesis, APCs senescence and its adverse outcomes on metabolism and T2D risk.

### Supplementary Information

**Additional file 1: Table S1**: List of primer sequences. **Table S2**: GO Cellular component analysis. Cellular components analysis of the genes differentially enriched in H3K4me3 in the FDR group. The table shows the GO terms ranked based on p-value computed from the Fischer exact test and the Gene ID of the overlapping genes. **Fig. S1** Differentially enriched regions for H3K4me3 in the CTRL and FDR groups were identified and annotated. (a) The reads obtained from ChIP-Seq data analysis featured a significant mapping around the TSS of the promoter (orange bar) compared to the reference genome (blue bar; 21.3% for <=1000 bp promoter and 24.3% for <=2000 bp promoter). (b) The pie chart of the identified 2644 H3K4me3 DERs were equally distributed between the CTRL and FDR groups (1477 CTRL vs 1167 FDR). (c) Of 1477 and 1167 DERs identified in CTRL and FDR, 1051 and 797 genes were, respectively, annotated near the gene TSS (± 1000 bps). **Fig. S2** H3K4me3 enrichment on positive and negative control gene targets. H3K4me3 enrichment on the PPARγ, Cytochrome c oxidase subunit 4I1 (COX4I1), and Citrate synthase (CS) promoters. (a) The H3K4me3 enrichment on positive GAPDH-TSS and negative control regions MB-Ex2 was measured by ChIP-qPCR in APCs using H3K4me3 and IgG antibodies (IgG was used as negative immunoprecipitation control). (b - d) H3K4me3 enrichment on PPARγ, COX4I1 and CS promoters was measured by ChIP-qPCR in subcutaneous APCs from FDR (n = 7) and CTRL (n = 9). (b - d) Data are shown as boxplots (min-max). (a - d) Statistical significance between the two groups was determined by an unpaired Student's t-test [ **p < 0.01, MB-Ex 2 vs GAPDH-TSS (a); *p < 0.05 vs CTRL (b - d)]. **Fig. S3** Schematic representation of the DNA methylation analysis performed on the TFAM promoter CpG island identified by Emboss Cpgplot. Methylation of all 50 CpGs at the TFAM promoter for ten replica clones. White circles, unmethylated CpGs; black circles, methylated CpGs. A representative experiment with one FDR and one CTRL individual is shown.

**Additional file 2. **Peaks annotation file in CTRL individuals. Genes (1051) annotated near the gene TSS (± 1000 bps), using the PAVIS tool, differentially enriched in CTRL subjects.

**Additional file 3. **Peaks annotation file in FDR individuals. Genes (797) annotated near the gene TSS (± 1000 bps), using the PAVIS tool, differentially enriched in FDR subjects.

## Data Availability

The raw data generated during the current study are available from the corresponding author on reasonable request. All analyzed data generated during this study are included in this published article [electronic supplementary material].
