# Supplementary material for: Altered H3K4me3 profile at the TFAM promoter causes mitochondrial alterations in preadipocytes from first-degree relatives of type 2 diabetics
Source: Clin Epigenetics. 2023 Sep 7;15:144. doi: 10.1186/s13148-023-01556-z (PMC10486065; doi:10.1186/s13148-023-01556-z)
Supplement: Supplementary file 1 — Additional file 1: Table S1: List of primer sequences. Table S2: GO Cellular component analysis. Cellular components analysis of the genes differentially enriched in H3K4me3 in the FDR group. The table shows the GO terms ranked based on p-value computed from the Fischer exact test and the Gene ID of the overlapping genes. Fig. S1 Differentially enriched regions for H3K4me3 in the CTRL and FDR groups were identified and annotated. (a) The reads obtained from ChIP-Seq data analysis featured a significant mapping around the TSS of the promoter (orange bar) compared to the reference genome (blue bar; 21.3% for <=1000 bp promoter and 24.3% for <=2000 bp promoter). (b) The pie chart of the identified 2644 H3K4me3 DERs were equally distributed between the CTRL and FDR groups (1477 CTRL vs 1167 FDR). (c) Of 1477 and 1167 DERs identified in CTRL and FDR, 1051 and 797 genes were, respectively, annotated near the gene TSS (± 1000 bps). Fig. S2 H3K4me3 enrichment on positive and negative control gene targets. H3K4me3 enrichment on the PPARγ, Cytochrome c oxidase subunit 4I1 (COX4I1), and Citrate synthase (CS) promoters. (a) The H3K4me3 enrichment on positive GAPDH-TSS and negative control regions MB-Ex2 was measured by ChIP-qPCR in APCs using H3K4me3 and IgG antibodies (IgG was used as negative immunoprecipitation control). (b - d) H3K4me3 enrichment on PPARγ, COX4I1 and CS promoters was measured by ChIP-qPCR in subcutaneous APCs from FDR (n = 7) and CTRL (n = 9). (b - d) Data are shown as boxplots (min-max). (a - d) Statistical significance between the two groups was determined by an unpaired Student's t-test [ **p < 0.01, MB-Ex 2 vs GAPDH-TSS (a); *p < 0.05 vs CTRL (b - d)]. Fig. S3 Schematic representation of the DNA methylation analysis performed on the TFAM promoter CpG island identified by Emboss Cpgplot. Methylation of all 50 CpGs at the TFAM promoter for ten replica clones. White circles, unmethylated CpGs; black circles, methylated CpGs. A representative experiment wit [file 13148_2023_1556_MOESM1_ESM.pdf]

Table S1. List of primer sequences.

Assay	Gene name	Primer sequence (5' to 3')
**qPCR primers**	***TFAM***	F: TGGCGTTTCTCCGAAGCAT<br>R: ACACAAAACTGAAGGGGGAGC
	PPARγ	F: TCTCACCTGGAAGACAGCTCC<br>R: GCTGATGATCATGTTGGGCTTGG
	GLUT4	F: CAGAAGGTGATTGAACAGAG<br>R: AATGATGCCAATGAGAAA
	ADIPOQ	F: GTGACGACACCAAAAGGGCTC<br>R: TCCAACCTGCACAAGTTCCC
	COX4I1	F: CAGCCAGAAGGCATTGAAGG<br>R: GGGCCGTACACATAGTGCTTC
	CS	F: TTCCGACCCTTACCTGTCCT<br>R: ATAGCCTGGAACAACCCGTC
	NRF1	F: ATGTCCGCACAGAAGAGCAA<br>R: TTCCCGCCCATGCTGTTTAT
	NRF2	F: GGACCTCACACTTCTAGTCGC<br>R: CAAAGGATCAGTCCCACTCCG
	PGC1α	F: TGACTGGCGTCATTCAGGAG<br>R: AACCAGAGCAGCACACTCG
	CYTB	F: TCTCCGATCCGTCCCTAACA<br>R: GTGATTGGCTTAGTGGGCGA
	CO2	F: TCCCGATTGAAGCCCCCATTC<br>R: GGCATGAAACTGTGGTTTGCTCC
	ATP6	F: AGGCACACCTACACCCCTTA<br>R: GATATTGCTAGGGTGGCGCT
	ND1	F: AACATACCCATGGCCAACCT<br>R: AGCGAAGGGTTGTAGTAGCCC
	TBP	F: GCCGGCTGTTTAACTTCGCT<br>R: GAAACAGTGATGCTGCTGGGT
	CDKN1A	F: GCAGACCAGCATGACAGATTTC<br>R: ATGTAGAGCGGGCCTTTGAG
	ZMAT3	F: TATCGAAGGGAGGGGAGCAA<br>R: TTAAAGGAGCCCATCTGCGG
**mtDNA content primers**	***HBB***	F: GAAGAGCCAAGGACAGGTAC<br>R: CAACTTCATCCACGTTCACC
	***tRNA* (*Leu*)**	F: CACCCAAGAACAGGGTTTGT<br>R: TGGCCATGGGTATGTTGTTA

**Bisulphite sequencing primers**	***TFAM***	F: GTTTTAAATGTGGTTTTAAGATTAT<br>R: GGATGTTAGTAGATTTTTTATAGTG
**Chip-qPCR primers**	***TFAM***	F: CGGGGTTGGAATGTAGACCC<br>R: AAGGTCGCGGTCTGAATCAA

Table S1. List of primers used in this study, related to the experimental procedures.

GO term	p-value	Odds Ratio	Combined score	Overlap genes
intracellular organelle (GO:0043229)	0.001700	1.37	8.72	[POP7, CCNT2, TFRC, ZFYVE9, WIPF1, CSE1L, NAB1, RPP30, SMC3, PSMD9, GOLGA3, RASSF1, FAM110B, EPC1, EP300, HADH, ACAA1, MED1, GABARAPL2, PRKCI, STX8, VPS33B, CLPX, THAP1, OXA1L, SUB1, WDFY1, TFAM, OPTN, IDI1, ANP32A, FLII, COPB1, NLK, ATP5B, NCSTN, BRIP1, DPP8, STK38, SCAP, MARK4, ZKSCAN1, TXNDC5, VASP, ANKRD27, TMEM50A, TRAPPC4, SETD1B, CDC7, SEPSECS, CDK7, ALDH6A1, ZBTB6, CDK4, BLMH, ZNF134, BCL2L1, RAD9A, SMG1, DAZAP1, DYRK3, ERO1LB, COX4I1, CTSZ, ARHGAP1, HSPB1, SIX1, IDE, ECSIT, USE1, GLI3, CHAF1B, TMEM43, MTERFD1, MRPL9, KDM6A, MKL2, H2AFX, SIRT5, TET1, AEN, RHOF, TERF1, GNL2, ARID1A, SLC39A14, ZHX1, CTTN, KIT, TOP1, PAFAH1B1, SMARCD1, WRB, DERL1, TTK, SDAD1, RNF4, PPP1R8, STX6, MTA2, EXOSC1, HNRNPA3, DUT, SLC35A3, YIPF2, EYA3, LIMK2, ERAP1, CTNS, MPP5, PEX13, APTX, ZNF33A, BCKDK, XAB2, MPHOSPH9, ZNF71, NMNAT1, ERCC4, DNAJA3, KLHL7, FAU]
mitochondrial part (GO:0044429)	0.002309	2.19	13.27	[TIMM8B, LRRC59, MRPS36, MRPL27, DLST, ETFB, MRPL13, CLPX, CS, BCKDK, ATP5B, OXA1L, DNAJA3, VDAC3, TFAM, MRPL9, HADH, RAB11FIP5, BCL2L1]
mitochondrial nucleoid (GO:0042645)	0.002648	5.98	35.50	[LRRC59, ATP5B, DNAJA3, TFAM, CLPX]
early endosome (GO:0005769)	0.003584	5.52	31.10	[ANKRD27, ZFYVE9, STX6, WDFY1, CTNS]
nucleoplasm (GO:0005654)	0.004047	1.95	10.75	[SMARCD1, RFC3, DUSP3, CCNT2, RFC4, OGG1, H2AFX, TAF11, CDC7, AEN, TERF1, GTF2F1, APTX, XAB2, CHAF1B, CDK7, CDK4, ERCC4, EPC1, TAF6, TOP1, E2F4]
cytosol (GO:0005829)	0.004356	1.62	8.83	[IDI1, ZFYVE9, YWHAB, SHMT1, COPB1, AKAP7, PIK3R1, CBL, HBXIP, CHAF1B, MAPK8, TBK1, AASDHPPT, GABARAPL2, ARFGEF2, PRKCI, DUT, HMGCS1, CSNK1A1, RIPK2, KIDINS220, DHPS, ERAP1, STAM, UBE2G2, RPS26, FKBP1A, CACNB3, RPS28, EIF3K, CDK4, PACS1, DNAJA3, WDFY1, GRB2, MVD, FAU, COPG, PAFAH1B1]
intracellular membrane-bounded organelle (GO:0043231)	0.007203	1.33	6.56	[POP7, CCNT2, TFRC, ZFYVE9, WIPF1, CSE1L, NAB1, RPP30, SMC3, PSMD9, GOLGA3, RASSF1, FAM110B, EPC1, EP300, HADH, ACAA1, MED1, GABARAPL2, PRKCI, STX8, VPS33B, CLPX, THAP1, OXA1L, WDFY1, TFAM, OPTN, IDI1, ANP32A, COPB1, ATP5B, NCSTN, BRIP1, DPP8, STK38, SCAP, ZKSCAN1, TXNDC5, ANKRD27, TMEM50A, TRAPPC4, CDC7, SEPSECS, CDK7, ALDH6A1, ZBTB6, CDK4, BLMH, ZNF134, BCL2L1, RAD9A, SMG1, DYRK3, ERO1LB, COX4I1, CTSZ, ARHGAP1, IDE, ECSIT, USE1, GLI3, CHAF1B, TMEM43, MTERFD1, KDM6A, MKL2, H2AFX, SIRT5, TET1, TERF1, GNL2, ARID1A, SLC39A14, ZHX1, KIT, TOP1, SMARCD1, WRB, DERL1, RNF4, PPP1R8, STX6, DUT, SLC35A3, YIPF2, LIMK2, EYA3, ERAP1, CTNS, MPP5, PEX13, ZNF33A, BCKDK, XAB2, MPHOSPH9, NMNAT1, ERCC4, DNAJA3]

Table S2. GO Cellular component analysis. Cellular components analysis of the genes differentially enriched in H3K4me3 in the FDR group. The table shows the GO terms ranked based on p-value computed from the Fischer exact test and the Gene ID of the overlapping genes.

Figure S1

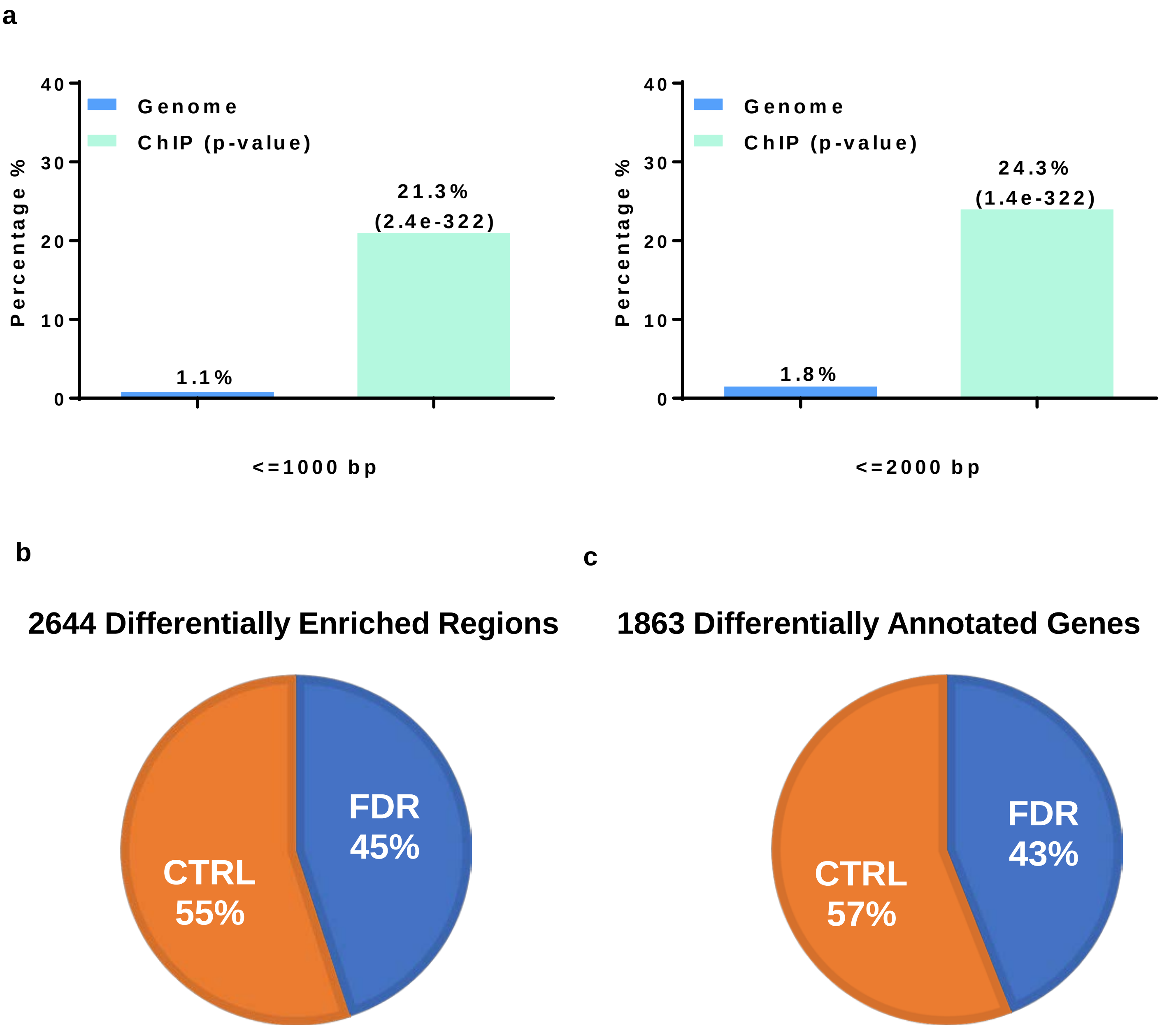

Fig. S1 Differentially enriched regions for H3K4me3 in the CTRL and FDR groups were identified and annotated. (a) The reads obtained from ChIP-Seq data analysis featured a significant mapping around the TSS of the promoter (orange bar) compared to the reference genome (blue bar; 21.3% for <=1000 bp promoter and 24.3% for <=2000 bp promoter). (b) The pie chart of the identified 2644 H3K4me3 DERs were equally distributed between the CTRL and FDR groups (1477 CTRL vs 1167 FDR). (c) Of 1477 and 1167 DERs identified in CTRL and FDR, 1051 and 797 genes were respectively annotated near the gene TSSs (± 1000 bps)

Figure S2

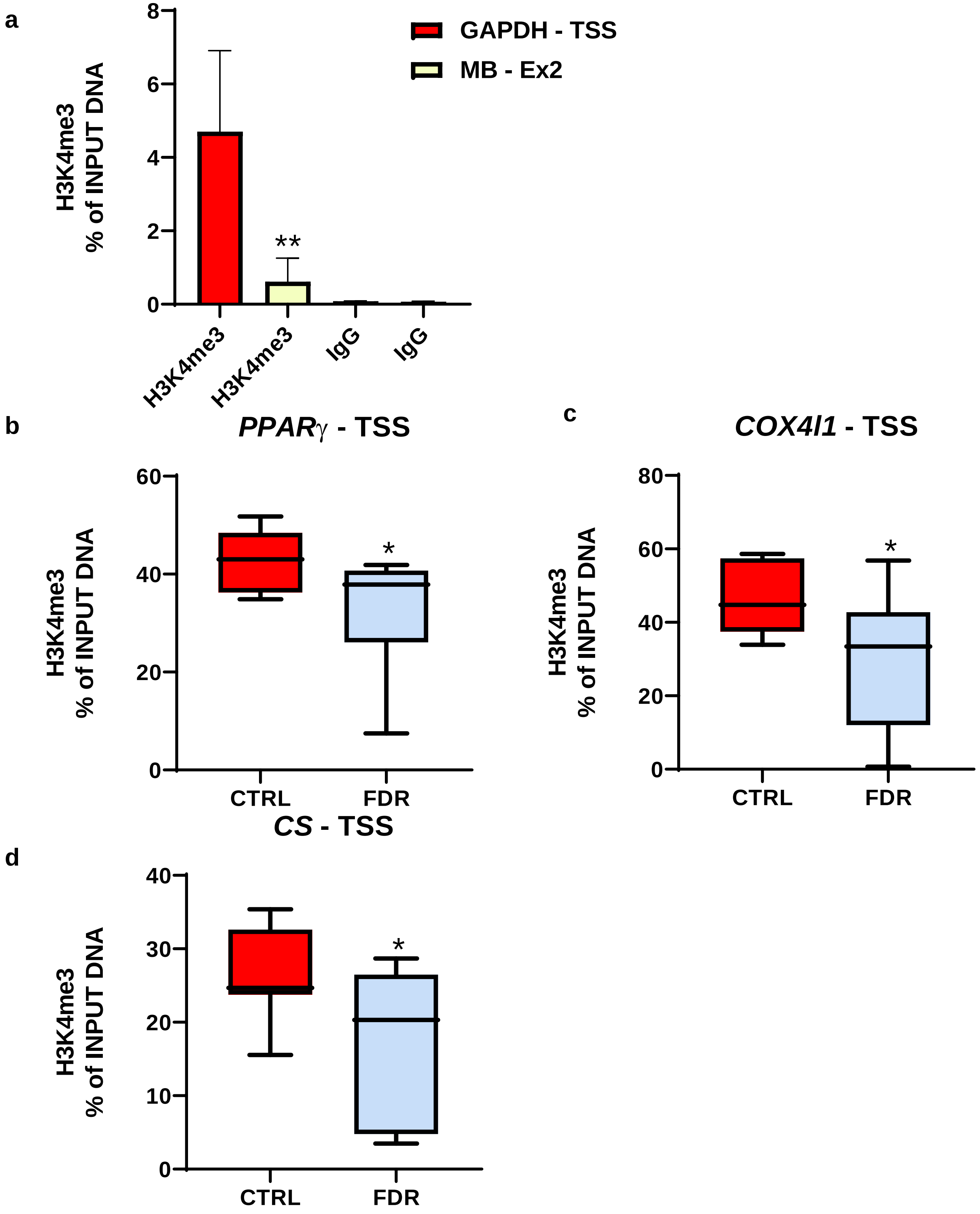

Fig. S2 H3K4me3 enrichment on positive and negative control gene targets. H3K4me3 enrichment on the *PPARγ*, *Cytochrome c oxidase subunit 4I1*, and *Citrate synthase* promoters. (a) The H3K4me3 enrichment on positive *GAPDH-TSS* and negative control regions *MB-Ex2* was measured by ChIP-qPCR in APCs using H3K4me3 and IgG antibodies (IgG was used as negative immunoprecipitation control). (b - d) H3K4me3 enrichment on *PPARγ*, *COX4I1* and *CS* promoters was measured by ChIP-qPCR in subcutaneous APCs from FDR (n = 7) and CTRL (n = 9). (b - d) Data are shown as boxplots (min-max) with all individual values. (a - d) Statistical significance between the two groups was determined by an unpaired Student's t-test [$^{**}p < 0.01$, MB-Ex 2 vs GAPDH-TSS (a); $^{*}p < 0.05$ vs CTRL (b - d)]

Figure S3

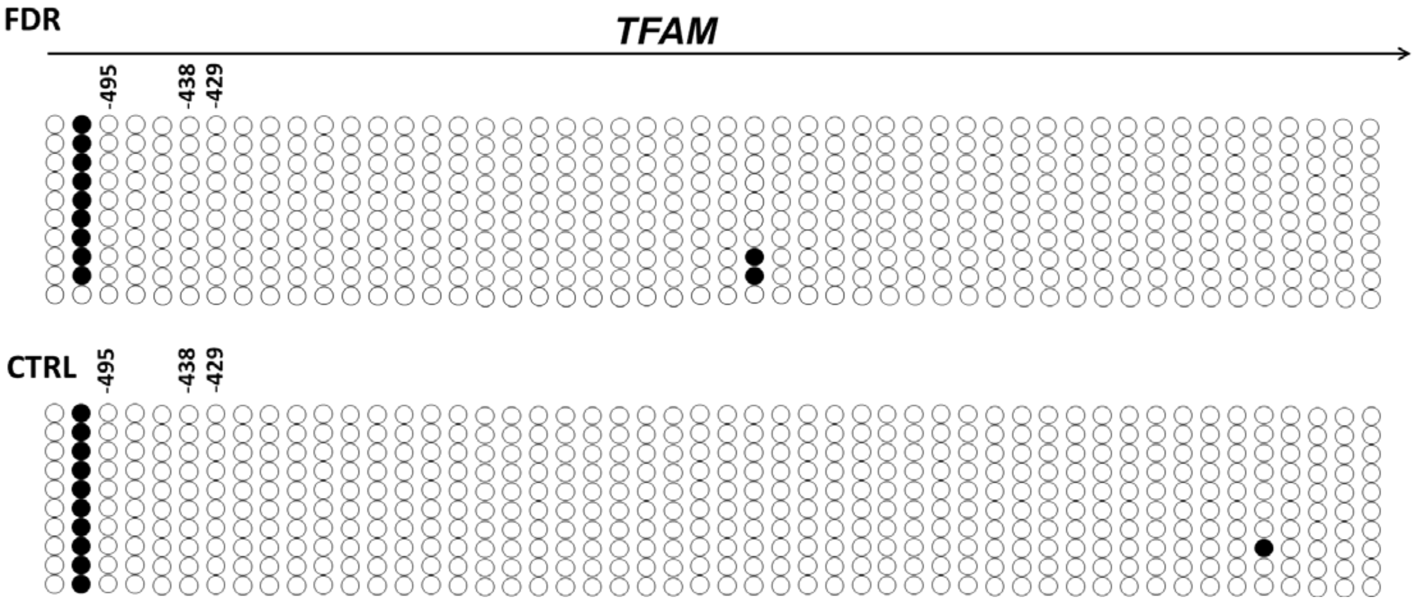

Fig. S3 Schematic representation of the DNA methylation analysis performed on the *TFAM* promoter CpG island identified by Emboss Cpgplot. Methylation of all 50 CpGs at the *TFAM* promoter for ten replica clones. White circles, unmethylated CpGs; black circles, methylated CpGs. A representative experiment with one FDR and one CTRL individual is shown
